# Injured inflammatory environment overrides the *TET2* shaped epigenetic landscape of pluripotent stem cell derived human neural stem cells

**DOI:** 10.1038/s41598-024-75689-3

**Published:** 2024-10-24

**Authors:** Noriko Kamei, Kenneth Day, Wei Guo, Daniel L. Haus, Hal X. Nguyen, Vanessa M. Scarfone, Keith Booher, Xi-Yu Jia, Brian J. Cummings, Aileen J. Anderson

**Affiliations:** 1 Sue & Bill Gross Stem Cell Research Center, University of California, Irvine, Irvine, CA 92697-1705 USA; 2 Zymo Research Corp, 17062 Murphy Ave, Irvine, CA 92614 USA; 3 Anatomy and Neurobiology, University of California, Irvine, Irvine, CA 92697-4475 USA; 4 Vidium Animal Health, 7201 E Henkel Way Suite210, Scottsdale, AZ 85255 USA

**Keywords:** Epigenetics, Neural stem cells, Epigenomics, Spinal cord injury

## Abstract

Spinal cord injury creates an inflammatory microenvironment that regulates the capacity of transplanted human Neural Stem Cells (hNSC) to migrate, differentiate, and repair injury. Despite similarities in gene expression and markers detected by immunostaining, hNSC populations exhibit heterogeneous therapeutic potential. This heterogeneity derives in part from the epigenetic landscape in the hNSC genome, specifically methylation (5mC) and hydroxymethylation (5hmC) state, which may affect the response of transplanted hNSC in the injury microenvironment and thereby modulate repair capacity. We demonstrate a significant up-regulation of methylcytosine dioxygenase 2 gene (*TET2*) expression in undifferentiated hNSC derived from human embryonic stem cells (hES-NSC), and report that this is associated with hES-NSC competence for differentiation marker expression. TET2 protein catalyzes active demethylation and *TET2* upregulation could be a signature of pluripotent exit, while shaping the epigenetic landscape in hES-NSC. We determine that the inflammatory environment overrides epigenetic programming in vitro and in vivo by directly modulating *TET2* expression levels in hES-NSC to change cell fate. We also report the effect of cell fate and microenvironment on differential methylation 5mC/5hmC balance. Understanding how the activity of epigenetic modifiers changes within the transplantation niche in vivo is crucial for assessment of hES-NSC behavior for potential clinical applications.

**Supplementary Information:**

The online version contains supplementary material available at 10.1038/s41598-024-75689-3.

## Introduction

Preclinical studies in animal models demonstrate that neural stem cell (NSC) transplantation can drive repair and recovery of function in central nervous system (CNS) injury and disease. Multipotent human NSC (hNSC) that retain self-renewal and in vitro expansion potential can be derived from pluripotent human embryonic stem cells (hESC) and human induced pluripotent stem cells (hiPSC). Prediction of cell line performance in the transplantation microenvironment is a key issue for development of cell-based clinical interventions. While a number of developmental and epigenetic regulatory mechanisms in NSC and brain neurogenesis have been described^1,2^, the regulation of epigenetic marks in hES-NSC/hiPS-NSC by exposure to different microenvironments remains a gap in our current knowledge.

Epigenetic changes, including DNA and histone modifications, drive key regulatory mechanisms of gene expression during brain development. DNA methylation (5mC) can be actively reversed by methylcytosine dioxygenase ten-eleven translocation (TET) 1, 2, and 3, enzyme activity via a hydroxymethylation (5hmC) intermediate^3,4^. The regulatory role of the TET family in neurogenesis is an active area of investigation^5,6^. The presence of 5hmC marks are most abundantly detected in brain^7–9^ and are stably maintained during early development, accumulating in postmitotic neurons^10,11^. Thus, active epigenetic modifications could play a significant role in gene regulation in hNSC during development, in tissue-derived fetal hNSC, and in hNSC created by the neuralization of pluripotent stem cells (PSC)^12,13^. However, the influence of intrinsic and extrinsic factors on the hNSC epigenome and gene expression is largely unknown.

Here, we tested the impact of both differentiation and the extrinsic inflammatory niche associated with CNS injury on the epigenome of PSC-derived hNSC using in vitro and in vivo models. Consistent with the premise that *TET* family genes play key roles in neurogenesis, neuralized-PSC exhibited up-regulation of *TET2* expression. Investigation of the epigenetic landscape of neuralized-PSC primed to different stage-specific lineage propensities (glial vs. neuronal) revealed changes in differential levels of methylation loci (5mC; DML) and hydroxymethylation loci (5hmC; DHML) patterns towards differentiation. Finally, analysis of neuralized-PSC re-isolated from the injured spinal cord (SC) after transplantation identified in vivo injury-associated microenvironmental influences on the epigenetic landscape and cell fate.

## Results

### *TET2* is significantly up-regulated in hNSC derived from pluripotent stem cells

**Fig. 1:**
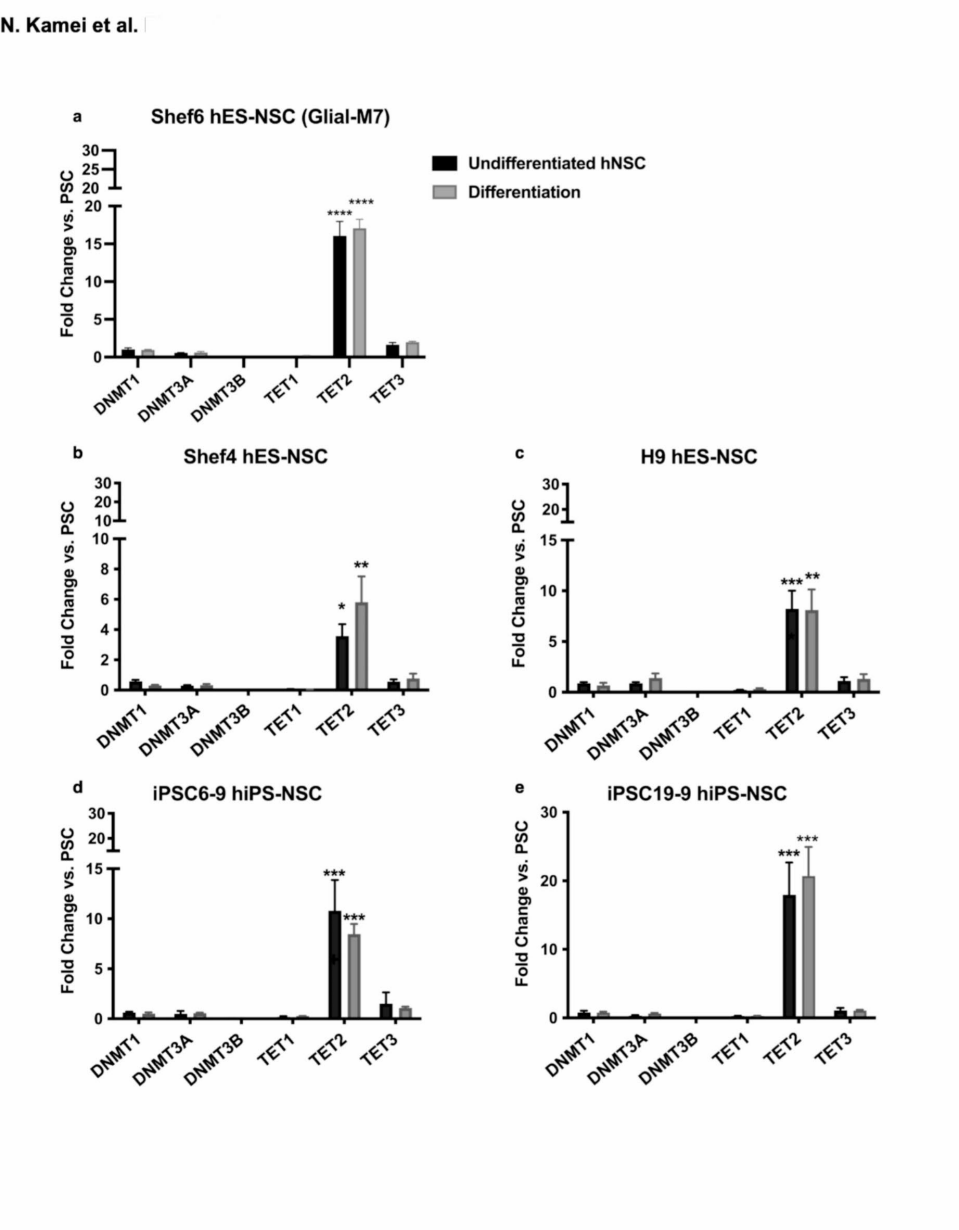
*TET2* gene expression is significantly up-regulated in hES-NSC/hiPS-NSC compared to hESC/hiPSC. Comparison of the fold change in expression of DNA Methyltransferase gene (*DNMT1*, *3 A*, and *3B*) and Methylcytosine dioxygenase gene (*TET1*, *2*, and *3*) are shown for different cell lines: (**a)**: Shef6 derived hES-NSC (Glial-M7) ; **(b)**: Shef4 derived hES-NSC; **(c)**: H9 derived hES-NSC (**d)**: iPS 6–9 derived hiPS-NSC; (**e)**: iPS 19−9 derived hiPS-NSC. Each bar shows undifferentiated hES-NSC or hiPS-NSC (dark grey) and 2 weeks differentiated hES-NSC or hiPS-NSC (grey) under normal neural differentiation media (DM). All data were normalized to the internal control 18 S rRNA and analyzed based on the expression level of each gene in each cell line pluripotent stem cells. Error bars represent standard error of the Mean (SEM; *n* = 3). Statistical analysis by one-way ANOVA with post-hoc Tukey *: *P* < 0.05, **: *P* < 0.005, ***: *P* < 0.001, ****: *P* < 0.0001 (hES-NSC/hiPS-NSC or Differentiated vs. hESC/hiPSC) (*n* = 3).

We sought to compare changes in the expression of enzymes regulating DNA methylation and demethylation in human embryonic and induced PSC lines (hES, hiPS) in comparison with neuralized derivatives (hES-NSC, hiPSC-NSC). Neuralized derivative lines were generated in parallel under the same protocol^14,15^, and were verified to exhibit multipotency in vitro via immunocytochemical and gene expression analysis of neural, astrocyte, and oligodendroglial lineages (Supplementary Fig.S1)^14,15^. We analyzed the expression of the DNA methyltransferase (DNMT) family genes *DNMT1*, *3 A*, and *3B*, and methylcytosine dioxygenase ten-eleven translocation (TET) family genes *TET1*,* 2*, & *3*. *DNMT* genes catalyze methylation of unmethylated cytosine on DNA. *TET* genes catalyze the demethylation of methylated cytosine to hydroxymethylated cytosine on DNA. For these analyses, we conducted quantitative PCR (qPCR), normalized by 18 S rRNA expression. Figure 1 shows fold-change expressed relative to the PSC parental line, demonstrating both *TET2* expression up-regulation in all derivative undifferentiated hNSC lines, and line-to-line variation in the magnitude of this up-regulation (Fig. 1a-e, black-bars). Up-regulation of *TET2* expression was retained in all lines when hNSC was cultured in differentiation media (DM; Fig. 1a-e; grey-bars). *TET2* up-regulation in neuralized-PSC derivatives was validated using a second protocol (Supplementary Fig.S2).

**Fig. 2:**
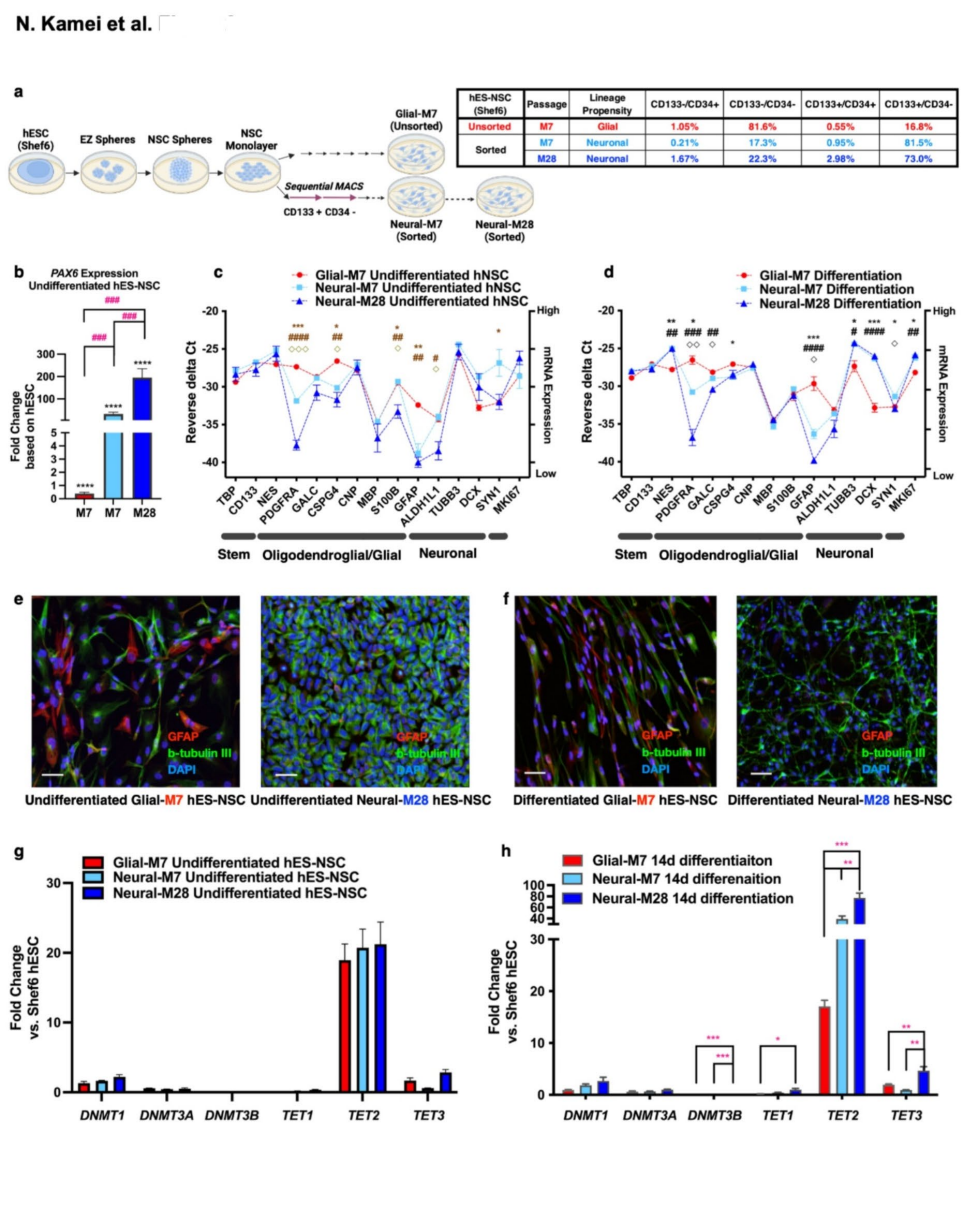
Relationship of Glial-M7, Neural-M7, and Neural-M28 hES-NSC, which are all derived from hESC, Shef6. The schematic diagram (**a-left**) shows the process of the derivation and subsequent neuralization of hES-NSC for Glial-M7, Neural-M7, and Neural-M28 from hESC (Shef6) via the EZ sphere-based protocol. All cell lines are derived from monolayer NSC. Neural-M7 and -M28 are passaged after Magnetic-Activated Cell Sorting (MACS) for the positive selection of CD133 + cells (retain) and negative selection of CD34 + cells (retain CD34- population) sequentially from monolayer-NSCs. We used a negative selection of CD34 to eliminate multiple cell types expressing CD34 including hematopoietic stem cells, endothelial cells, macrophages, and microglia populations^15^. Table (**a-right**) shows the representative percentage of each population (CD133+/CD34+, CD133+/CD34-, CD133-/CD34+, CD133-/CD34-) that exist in Glial-M7 (Red), Neural-M7 (Light Blue), and Neural-M28 (Dark Blue) hES-NSC after sorting or without sorting for two cell surface markers: CD133 and CD34, and analyzed by the FACS. (**b**) *PAX6* expression in undifferentiated hES-NSC compared to the expression level in Shef6 hESC are shown. Selected gene panel under undifferentiated stage (**c**) and normal differentiation (DM) conditions in vitro (**d**) among Glial-M7, Neural-M7 & M28 cells are shown. [one-way ANOVA post- hoc Tukey test: M7 vs. M28: **#***p* < 0.05, **##***p* < 0.005, **####***p* < 0.0001 (Undifferentiated) & **#***p* < 0.05, **##***p* < 0.005, **###***p* < 0.0005, **####***p* < 0.0001 (Differentiation), M7 vs. M7: *p < 0.05, **p < 0.005, ***p < 0.0005 (Undifferentiated) & *p < 0.05, **p < 0.005, ***p < 0.0005 (Differentiation), M7 vs. M28: ◇*p* < 0.05, ◇◇◇*p* < 0.0005 (Undifferentiated) & ◇*p* < 0.05, ◇◇*p* < 0.005, (Differentiation).] The dotted lines shown in (c) and (d) are only for visual purposes and do not imply direct relationships. Immunocytochemistry of Glial-M7 and Neural-M28 detected with GFAP (red), Beta-tubulin (green), and nuclei with Hoechst 33342 (blue) are shown for Undifferentiated (**e**) and 14 DIV differentiation (**f**). The qPCR results are shown for fold change of gene expressions for *DNMT* (*1*,* 3 A*, and *3B*) and *TET* (*1*,* 2*, and *3*) at undifferentiated (**g**) and differentiated for 14 DIV (**h**). The lineage marker genes are shown on the X-axis of (**c**) and (**d**): each horizontal bar represents Stemness/proliferation-related, oligodendrocytes-related, Glial cell-related, neuron-related, and proliferation-related genes. Error bars represent standard error (*n* = 3). Statistical analysis by one-way ANOVA with post-hoc Tukey **** *p* < 0.0001 (vs. hESC), * *p* < 0.05, ** *p* < 0.005, *** *p* < 0.0005, or ### *p* < 0.0005 (among cell lines). The Scale bar in (**e**) & (**f**) is shown as 50 μm.

We hypothesized that variation in *TET2* expression regulation during differentiation could modulate the epigenetic landscape to prime lineage propensity. To minimize the potential contribution of variation observed between parent lines, we employed hES-NSC derivative lines previously generated from hESC (Shef6) using a xeno-free sphere-based neuralization protocol^15^. All hES-NSC derivative lines were propagated in monolayer (M) conditions and are identified as follows: Unsorted-M7 (referred-to-as Glial-M7), Sorted-M7/M28 (referred-to-as Neural-M7; Neural-M28) (Fig. 2a). Neural-M7 was generated from monolayer-NSC using magnetic-activated cell sorting (MACS) with positive selection for the cell surface stem cell marker CD133, as previously described^15^. Neural-M28 was propagated from monolayer passage Neural-M7 to M28 (Supplementary Fig.S3).

During development, radial glial cells divide asymmetrically to first generate neurons through intermediate progenitor cells, then glial cells, and eventually oligodendrocytes^16^. The method we used to create hES-NSC (Fig. 2a) is known to produce neural progenitors as well as radial glial marker-positive cells^17^. Glial-M7 contains such a radial glial cell population, thus having a glial propensity. However, Neural-M7 and Neural-M28, a CD133-positive enriched population of cells by sorting, may be augmented with early progenitor cells that create more neurons than Glial-M7, therefore having a neural propensity.

Both sorted and unsorted hES-NSC derivatives retained karyotype and multipotency^15^. This is consistent with previous demonstrations that CD133 deletion does not alter hES pluripotency or germ layer formation^18^, and that NSC potency is not exclusive to CD133 + populations. However, the lineage propensity of Glial-M7, Neural-M7, and Neural-M28 differed. Thus, these cell lines are multipotent hES-NSC but may represent cell populations at different stages of development. CD133-sorted, hES-NSC derivatives (Neural-M7 and Neural-M28) exhibited increased *PAX6* expression and a shift to a neuronal lineage preference in comparison with unsorted sister hES-NSC (Glial-M7; Fig. 2b, c,d). This shift was enhanced in Neural-M28 vs. Neural-M7 hES-NSC for some, but not all, markers. The shift was also confirmed via immunocytochemistry (Fig. 2e, f) for the neuronal marker beta-tubulin III (b-Tub III, Green) and astrocytic marker glial fibrillary acidic protein (GFAP, Red). Glial-M7, Neural-M7, and Neural-M28 exhibited comparable *TET2* up-regulation in comparison with the Shef6 hESC parental line (Fig. 2g). However, under differentiation conditions (DM), the CD133-sorted Neural-M7 cells exhibited enhanced upregulation of *TET2* vs. Glial-M7 cells, which was sustained with extended passage in Neural-M28 cells (Fig. 2h). These data suggest a relationship between *TET2* upregulation levels and neuronal lineage propensity. Based on these data, we selected Glial-M7 and Neural-M28 as tools to investigate the relationship between *TET2* regulation and lineage propensity.

### Gene knockdown (KD) modulation of *TET2* and *DNMT3A* in Glial-M7 and Neural-M28 hES-NSC

**Fig. 3:**
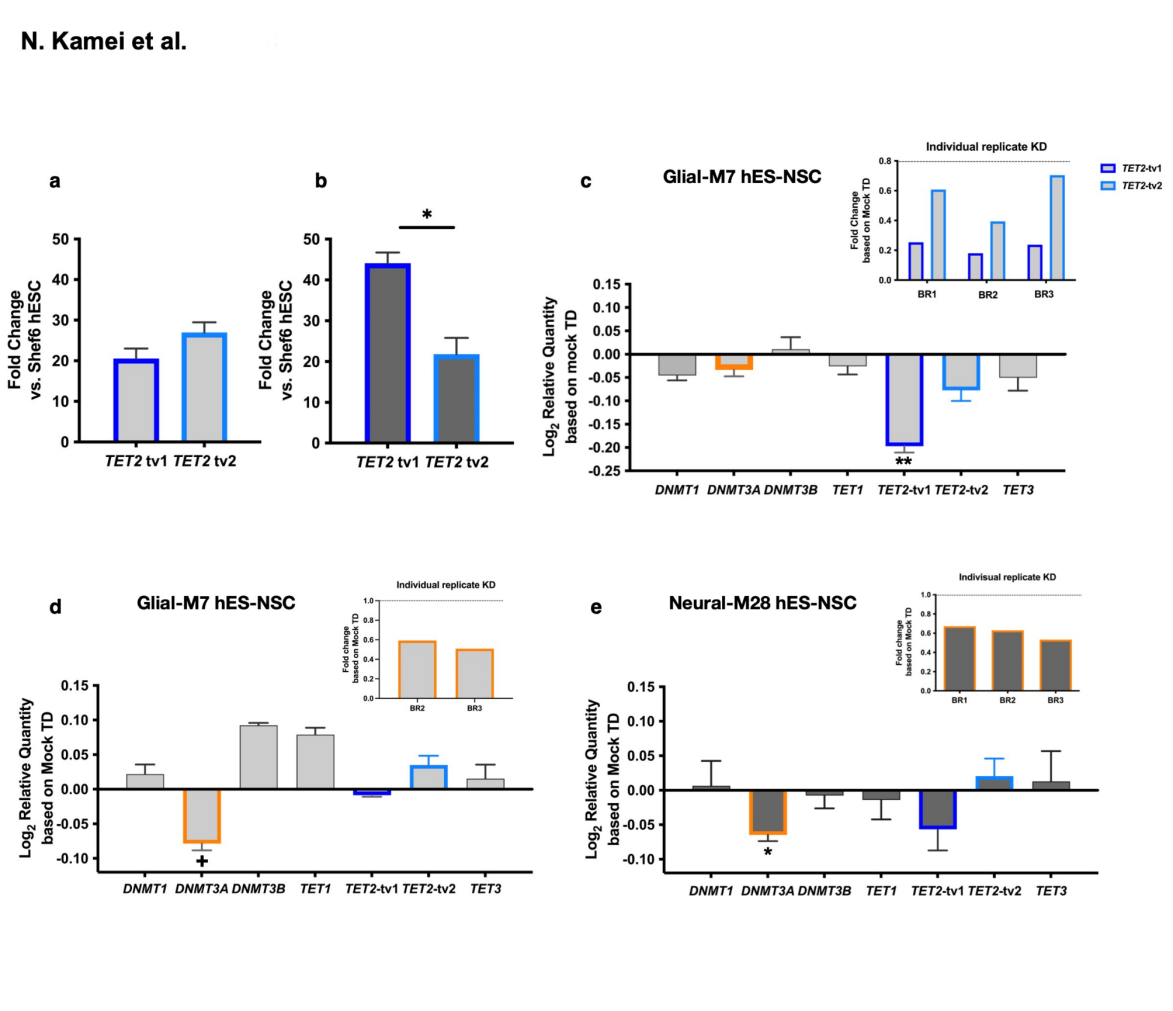
*TET2* gene Knockdown (KD) and *DNMT3A* gene KD performed in either Glial-M7 (light grey) or Neural-M28 (dark grey). Shown are different levels of *TET2* transcript variants (tv1 and tv2) gene expression detected in undifferentiated Glial-M7 (**a**) and Neural-M28 (**b**). *TET2* gene KD was shown in undifferentiated Glial-M7 (**c**), and *DNMT3A* gene KD was shown in undifferentiated Glial-M7 (**d**) or Neural-M28 (**e**). All KD results shown in this figure were analyzed only for reporter gene-positive cells (RFP), which is expressed with each shRNA on the bicistronic construct, by FAC sorting (BD science Aria II). Results of either Knockdown in either cell line are shown as transformed Log2 relative quantity of *DNMT* gene family (*DNMT1*, *3 A*, and *3B*) and *TET* gene family (*TET1*,* TET2*-tv1 and -tv2, and *TET3*) expression compared to cells with mock transduction (TD) controls. The fold change of each gene in KD cells was detected from the delta Ct as the gene expression level for each gene in KD cells based on the delta Ct of each gene expression level in Mock transduced cells. Then, we transform the fold change into the Log2 scale. Data represents *N* = 2 ~ 3 biological replicates (BR) as indicated (BR1, BR2, BR3), and the fold change of individual biological replicate’s result are shown in the inset. All error bars represent the standard error of the mean. Statistical analysis by one sample t-test vs. control (**: *P* < 0.005 * : *P* < 0.05, +: *P* < 0.08).

We sought to investigate the role of cytosine methylation on lineage preference using *TET2* depletion via transient gene knockdown (KD) with lentiviral shRNA transduction in undifferentiated cells. We verified KD efficiency with each lentiviral shRNA in undifferentiated Glial-M7 and Neural-M28 and pooled validated shRNAs to maximize KD. We also analyzed expression levels of the major transcript variants of *TET2* (tv1 and tv2) in Glial-M7 and Neural-M28; this analysis identified differences in *TET2* transcript variant usage (Fig. 3a, b) and could suggest distinct roles for the tv1 and 2 variants in neuronal and glial lineages.

While shRNA treatment achieved efficient *TET2* KD for Glial-M7 (Fig. 3c), stable *TET2* KD was not detected in Neural-M28 (Supplementary Fig.S4, S5). This result could reflect either a lack of durability of effect in the Neural-M28 due to the existence of *TET2* transcript variants, or unknown factors. Regardless, this finding necessitated an adjunct approach to probe the effect of cytosine methylation on lineage preference. *TET* genes catalyze the demethylation of methylated cytosine to hydroxymethylated cytosine on DNA. *DNMT* genes catalyze methylation of unmethylated cytosine on DNA. Because *TET2* KD was not stable in Neural-M28, we selected *DNMT3A* KD as an alternative means by which to probe the role of cytosine methylation status in lineage propensity. Knockdown of *DNMT3A*, which is also known to regulate neurogenesis, exhibited reproducible reduction in both Glial-M7 (Fig. 3d) and Neural-M28 (Fig. 3e). Unexpectedly, *TET2*-tv1 expression also exhibited a trend for decreased expression in *DNMT3A* KD Neural-M28 (Fig. 3e). This observation may suggest that a linkage in the regulation of *TET2* and *DNMT3A* establishes the epigenetic landscape in hES-NSC^19,20^.

### Knockdown of *TET2* or *DNMT3A* in undifferentiated hES-NSC alters cell differentiation and responses between normal and inflammatory microenvironments

Because both KD approaches exhibited sustained modifications of target gene expression in Glial-M7 hES-NSC, we analyzed the effect of both *TET2* and *DNMT3A* KD in these cells. In contrast, because *TET2* KD in Neural-M28 hES-NSC was not sustained, we restricted analysis to *DNMT3A* KD in this case.

**Fig. 4:**
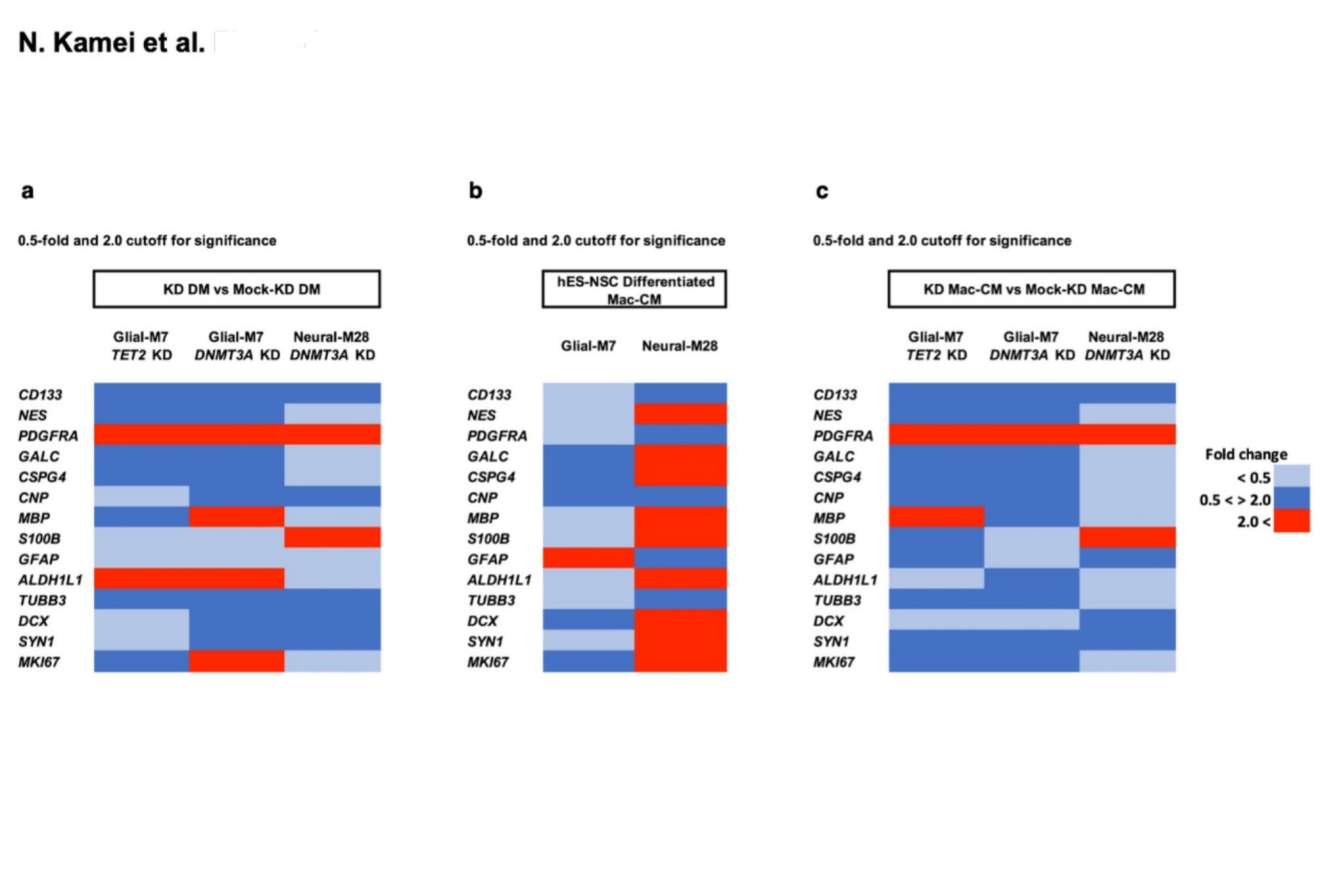
Heat maps of selected gene expression patterns that are changed after Glial-M7 or Neural-M28 hES-NSC transient KD of either *TET2* or *DNMT3A* differentiated under normal (DM) and inflammatory differentiation (Mac-CM) conditions. Differentiation gene expression profiles under DM (**a**) and Mac-CM (**c**) of Glial-M7 and Neural-M28 three days after gene Knockdown (KD) of either *TET2* or *DNMT3A* at the undifferentiated stage, as well as differentiation profiles under Mac-CM without KD (**b**), are shown. The same KD cells shown in Fig. 3(**c**) (*TET2* KD), Fig. 3(**d**) and (**e**) (*DNMT3A* KD) were spread and differentiated in two different conditions for 14 days and analyzed for reporter gene positive cells (RFP) only as explained in Fig. 3. The colors on the Heat maps showed the fold change data cut off below 0.5; down-regulated (Light-Blue), beyond 2.0; up-regulated (Red) for significant of gene expression of a selected gene panel compared to mock transduction control (Mock-KD) differentiation results. The fold change between 0.5 to 2.0 are shown in Blue.

We first established the baseline expression profile of a defined set of neural lineage genes for Glial-M7 and Neural-M28 hES-NSC in response to *TET2* and *DNMT3A* KD under DM conditions, identifying three categories of gene expression changes: (1) similar expression levels neither gene KD nor cell lines dependent (*CD133*, *PDGFRA*, *TUBB3*); (2) only cell line dependent (*ALDH1L1*, *S100B*); and, (3) both KD gene and cell line dependent (*MBP*,* MKI67*) (Fig. 4a). We have shown that there is a multiphasic inflammatory response after spinal cord injury (SCI)^21^. The chronic SCI microenvironment is heavily defined by macrophages/microglia^21^. We have previously employed exposure to macrophage-conditioned differentiation media (Mac-CM) as a strategy to define the effect of the inflammatory factors relevant to the in vivo microenvironment on hNSC in vitro^21^. Therefore, we next tested the effect of Mac-CM treatment during differentiation conditions^21^ on expression of these neural lineage genes, identifying striking differences in the effect of this inflammatory stimulus on gene expression between Glial-M7 and Neural-M28 cells (Fig. 4b). Neural-M28 exhibited upregulation of diverse lineage marker genes, while Glial-M7 exhibited down regulation of nearly all lineage marker genes except for *GFAP*. Lastly, we demonstrated that *DNMT3A* KD blocks the robust upregulation of neural lineage marker genes detected in Neural-M28 under the Mac-CM (Fig. 4c vs. 4b)^19,22,23^. Notably, the inflammatory environment provided by Mac-CM altered lineage marker expresion in  *TET2* or *DNMT3A* KD cells compared to DM (Fig. 4a vs. 4c); within each biological replicate, all cells were plated and analysed in parallel to enable valid comparison. These data suggest that the inflammatory microenvironment can override or alter epigenetic regulation in these cells. Thus, *TET2* and *DNMT3A* may act in a synergistic manner to regulate the balance of 5mC and 5hmC levels and/or at the particular regions on the genome to regulate or determine neural cell fate^19,22,24^. Together, these data indicate that the injured inflammatory microenvironment overrides the epigenetic landscape of *TET2* or *DNMT3A* KD hES-NSC, which could involve chromatin changes to alter gene expression. However, this effect was different depending on each hES-NSC initial epigenetic profile (Fig. 4).

### Differential levels and patterns of methylation (5mC) and hydroxymethylation (5hmC) in TF genes distinguish environmental exposure effects during NSC differentiation

**Fig. 5:**
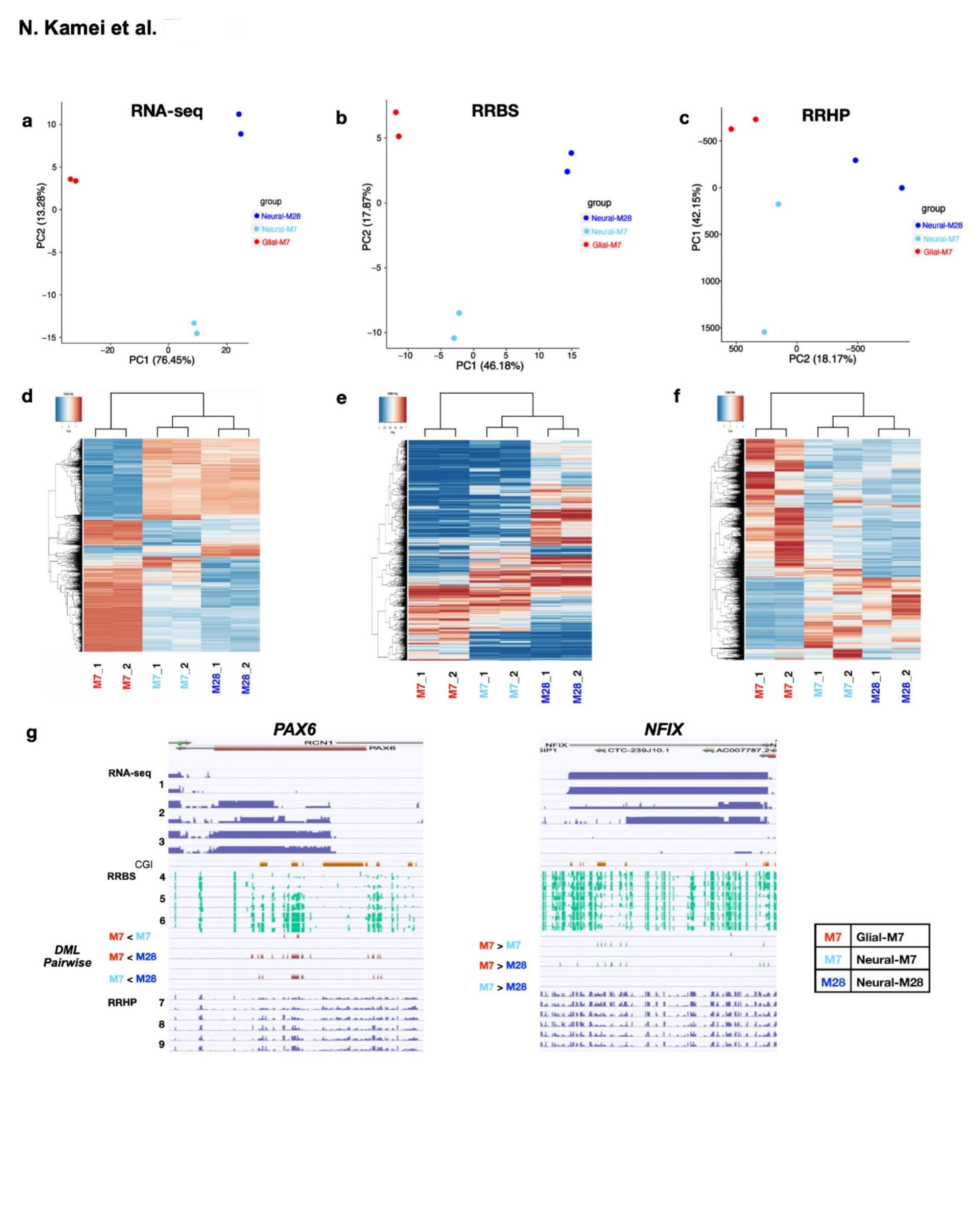
Genome-wide analyses of Undifferentiated Glial-M7, Neural-M7, and Neural-M28 for RNA-seq, Reduced Representation Bisulfite Sequencing (RRBS), and Reduced Representation Hydroxymethylation Profiling (RRHP) are shown. (**a**)-(**c**) show the principal component analysis results among three cell lines: Glial-M7, Neural-M7 and Neural-M28 for RNA-seq (**a**), RRBS (**b**), and RRHP (**c**). Red dots are Glial-M7, Light Blue dots are Neural-M7, and Blue dots are Neural-M28. (**d**)-(**f**) shows the hierarchical clustering results among Glial-M7, Neural-M7, and Neural-M28 for RNA-seq (**d**), RRBS (**e**), and RRHP (**f**). The column from the left shows two biological replicates, each of Glial-M7, Neural-M7, and Neural-M28. (**g**) Shows the absolute value of gene expression (RNA-seq), methylation (5mC, RRBS), and hydroxymethylation (5hmC, RRHP) detection levels among undifferentiated stage of Glial-M7: lane 1, 4, & 7, Neural-M7: lane 2, 5, & 8, and Neural-M28: lane 3, 6, & 9. Read data were mapped on the genome, and the exemplary relationships between 5mC/5hmC for higher gene expression towards Neural-M28 (*PAX6*; left) or towards Glial-M7 (*NFIX*; right) are shown (lane 1–9). Each lane with numbers 1–9 was constructed from the results of two biological replicates. Lane designations on the left apply for both *PAX6* and *NFIX*. Lanes 1–3 are for RNA-seq, lanes 4–6 are for RRBS, and lanes 7–9 are for RRHP. The differential methylation loci (DML) and the level of methylation differences from the pairwise analysis of Glial-M7, Neural-M7, and Neural-M28 are shown as DML pairwise analysis between lanes 6 and 7 in (**g**).

To directly examine 5mC and 5hmC (5mC/5hmC), we analyzed genome-wide differential 5mC/5hmC in undifferentiated Glial-M7, Neural-M7, and Neural-M28 cells by reduced representation bisulfite sequencing (RRBS)^25,26^ and reduced representation hydroxymethylation profiling (RRHP)^27^. Principal Component Analyses (PCA) (Fig. 5a-c) and hierarchical clustering with input RNA-seq normalized read counts, RRBS, and RRHP (Fig. 5d-f) comparing undifferentiated Glial-M7, Neural-M7, and Neural-M28 show a relatively concordant relationship between biological replicates.

The absolute value of gene expression (RNA-seq), methylation (5mC, RRBS), and hydroxymethylation (5hmC, RRHP) levels among undifferentiated Glial-M7, Neural-M7, and Neural-M28 cells were mapped on the genome (Fig. 5g; lane 1–9). The exemplary gene expression changes that increase towards Neural-M28 (*PAX6*), or Glial-M7 (*NFIX*) appear to correlate with the specific locations of 5mC/5hmC detection level changes identified in the genome among cell line derivatives. These differential levels of 5mC differences may reflect gene expression status and may also indicate priming in advance of activation for the next developmental stage since these data were collected from undifferentiated cells.

To probe the impact of the effect of the inflammatory environment on the 5mC/5hmC epigenetic landscape for Glial-M7, Neural-M7, and Neural-M28 cells during differentiation, we analyzed the differentially changed detection levels of methylated loci (DML) and hydroxymethylated loci (DHML) for comparison of Glial-M7 and Neural-M28 cells between and within cell line derivatives across the following conditions: (1) undifferentiated; (2) normal differentiation media (DM); and (3) macrophage-conditioned differentiation media (Mac-CM)^28,29^.

**Fig. 6:**
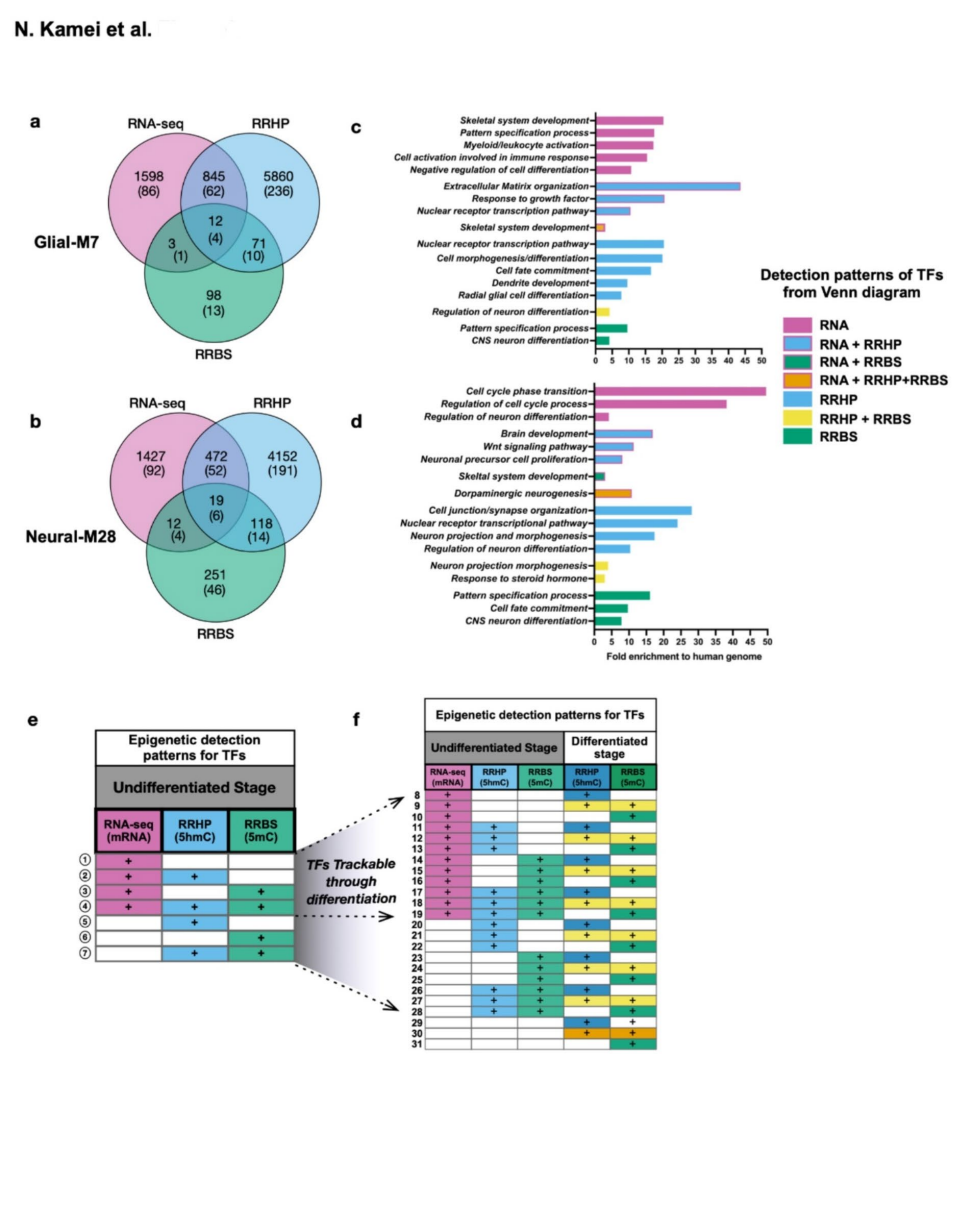
Epigenetic landscape of undifferentiated Glial-M7 and Neural-M28 by pairwise differential analysis of RNA-seq, 5mC (RRBS), or 5hmC (RRHP): epigenetic patterns change during differentiation. Venn diagrams show the number of annotated genes and transcription factor genes (each number in parenthesis) detected as significant by pairwise analysis of differential analysis of RNA-seq, 5mC, or 5hmC for and between Glial-M7 (**a**) and Neural-M28 (**b**) at the undifferentiated stage. Genes/transcription factor genes in (**a**) or (**b**) are detected with different epigenetic patterns for a combination of RNA-seq, RRBS, and RRHP. Representative biological processes identified for each pattern detected at the Undifferentiated stage for Glial-M7 (**a**) are shown in (**c**), and Neural-M28 (**b**) are shown in (**d**). Epigenetic patterns for each TF gene change from undifferentiated (**e**) to differentiated (**f**). The same transcription factor genes identified in each pattern with the pairwise differential analysis of RNA-seq, RRBS, or RRHP at the undifferentiated stage are shown in (**e**), and some TFs are also detected at the differentiated stage (**f**) for the pattern groups that increase 8–28; termed trackable (T). However, some transcription factor genes are only detected in either at undifferentiated (pattern 1–7; circled-number) or at differentiated stage (pattern 29–31) and were termed untrackable (UnT). The blank cells shown in (**e**) and (**f**) indicate “not detected.”

These analyses were refined to focus on comparison of cells employed for expression and KD analysis in Figs. 3 and 4. Since these cell line derivatives are of the same hESC origin and samples were collected from each biological replicate before and after treatment, this paradigm avoids the confounding factor of differential 5mC/5hmC patterns resulting from genetic variation. The number of annotated genes, including transcription factor genes (TFs) detected as significant by differential analysis for RNA-seq, RRBS, or RRHP between undifferentiated cell line derivatives are shown by the Venn diagram (Fig. 6a, b, Supplementary Table S2). We further focused on genes annotated as TFs because TFs play important roles during neurogenesis to control the expression of genes that regulate cell-lineage specification along differentiation/development, suggesting that their activity could be modulated by environmental signaling factors^30–32^.

It has been suggested that gene expression patterns are regulated by chromatin structure established partly by the level of 5mC and 5hmC, and their balance is important for fate regulation, as well as establishment of, and changes in, 5mC/5hmC levels. Such epigenetic patterns are thought to control gene expression that are programmed along with development^33–33^. Thus, we categorized TFs with different combinations of patterns of RNA-seq, 5mC, and 5hmC detected after each method’s differential analysis for each cell line derivative by pairwise comparison through differentiation in DM or Mac-CM. TFs detected in undifferentiated cell line derivatives were involved in characteristic biological processes (Fig. 6c, d). TFs detected in Glial-M7 show alternative biological processes like Myeloid/leukocyte activation (Fig. 6c). However, as neuralized cells, both undifferentiated Glial-M7 and Neural M28 may be primed for neurogenesis-related processes (Fig. 6c, d) as shown in Fig. 2. TFs with patterns identified from the differential analysis of 5mC (DML) and/or 5hmC (DHML), but not with RNA-seq, were also involved in neurogenesis related biological processes (Fig. 6c, d). There is a complex relationship between 5mC/5hmC levels/locations in the genome and gene expression, which makes identification of consensus patterns underlying gene expression based solely on 5mC and 5hmC detection impossible^1,36–39^. However, we hypothesize that these epigenetic marks could be primed in advance of activation or be involved in cell fate decisions and differentiation. Thus, we further examined TFs detected with the 5mC/5hmC epigenetic marks to track pattern changes throughout differentiation from comparisons of the pairwise differential detection within each cell line and between Glial-M7 and Neural-28.

Some of the annotated TFs detected in undifferentiated cells were also detected after 14 days in vitro differentiation (DIV), but the DML and DHML epigenetic patterns identified from pairwise comparison analysis were different (Fig. 6e, f). While 7 epigenetic patterns (circled-numbers) were detected for TFs in undifferentiated cells (Fig. 6e), some genes/TFs were detected differentially with only RNA-seq, or RRBS, or RRHP, and some were detected with combinations of two or three of these methods. The number of epigenetic patterns detected and trackable through differentiation expanded to an additional 24 (uncircled-numbers 8–31) (Fig. 6f). We reasoned that if these epigenetic marks allow us to identify and indicate primed cell fate related genes ahead of expression, then the TF pattern changes associated with the transition from undifferentiated to differentiated stages may be useful to follow differentiation status^40^. We therefore divided the detected TFs into two categories for further analysis (Fig. 6f, Supplementary Table S3): (1) trackable TFs, meaning that we were able to track the same TF with DML/DHML in undifferentiated and differentiated cells (T; uncircled-number 8–28); and (2) untrackable TFs, meaning that a TF was detected only in either undifferentiated or differentiated cells (UnT).

**Fig. 7:**
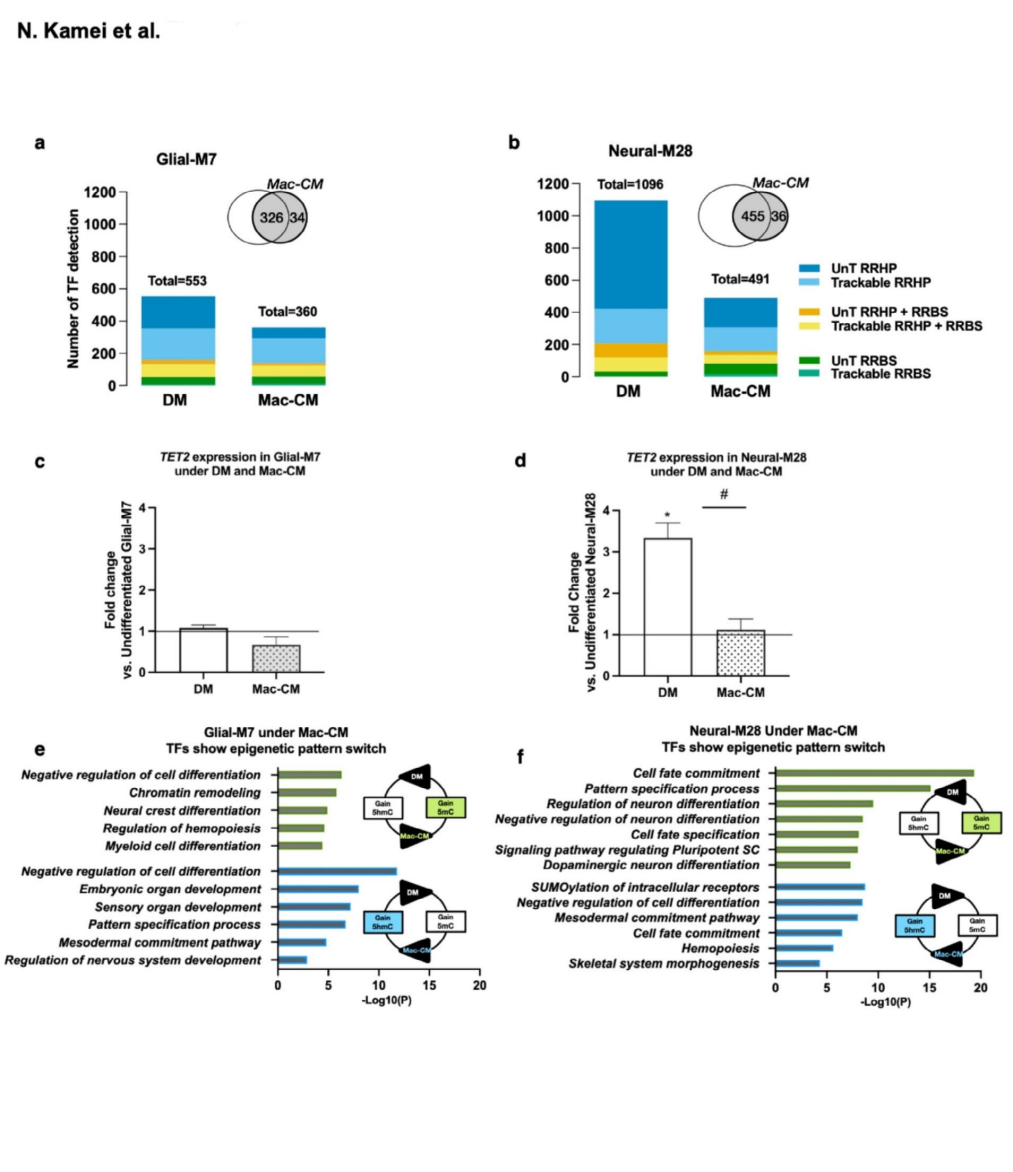
In vitro Inflammatory condition: Mac-CM directly affected *TET2* expression level in both Glial-M7 and Neural-M28. The graphs (**a**) and (**b**) show the number of TFs detected under normal (DM) and inflammatory (Mac-CM) differentiation conditions for (**a**) Glial-M7 or (**b**) Neural-M28. Each color represents the Transcription factor gene’s (TF) epigenetic pattern [Trackable (T): pattern 8 to 28 and Untrackable (UnT): pattern 29 to 31] shown in Fig. 6f. The graphs (**c**) and (**d**) show the fold change of *TET2* gene expression based on each undifferentiated hES-NSC under Normal (DM) or Inflammatory (Mac-CM) differentiation conditions media for Glial-M7 (**c**) and Neural-M28 (**d**). Statistical analysis by one-way ANOVA with post-hoc Tukey * *P* < 0.05 (vs. Undifferentiated); # *p* < 0.02 (DM vs. Mac-CM). Approximately 20–30% of the TF genes identified in both DM and Mac-CM in each cell line in (**a**) or (**b**) are involved in the same specific lineage pathways but altered with respect to the 5mC/5hmC patterns observed. The representative biological processes related to 5mC/5hmC pattern-switching TF genes under Mac-CM are shown for (**e**) Glial-M7 and (**f**) Neural-M28. Reciprocal epigenetic detection pattern for the same TF under Mac-CM has two groups: Green: Loss of 5hmC and Gain 5mC or Blue: Loss of 5mC and Gain of 5hmC (**e** and **f**). These 5mC and 5hmC profiles of the same TFs in (**e**) and (**f**) are flipped under DM.

We analyzed the changes of the 5mC/5hmC epigenetic patterns present for TFs for Glial-M7 vs. Neural-M28 following 14 DIV differentiation under DM or Mac-CM. The total number of TFs identified was different between DM and Mac-CM for both Glial-M7 (553 vs. 360) and Neural-M28 (1096 vs. 491). Two weeks of exposure to these different differentiation environments affected the overall number and level of significance of 5mC/5hmC (DML/DHML) mark detection on these TFs. For both Glial-M7 and Neural-M28, over 90% of TFs under Mac-CM overlapped with DM, which may be expected because these derivatives shared the same parent hES-NSC line which is already neuralized, and because Mac-CM shares a common media component base with DM. However, in contrast to Glial-M7, Neual-M28 cells exhibited a 3-fold upregulation of *TET2* under DM conditions, which was reversed in the presence of Mac-CM (Fig. 7c, d). A small but significant reduction of *TET2* expression levels under Mac-CM was also identified in Glial-M7 cells under the same conditions. Reduction of *TET2* expression levels observed under Mac-CM could be associated with the reduction in the number of TFs with significant changes detected during differentiation in the level of 5mC/5hmC (DML/DHML). Interestingly, *TET2* levels in Neural-M28 were consistently more elevated than those observed in Glial-M7 under both DM and Mac-CM.

Approximately 20–30% of the TF genes identified in both DM and Mac-CM in each cell line derivative are involved in the same specific lineage pathways but altered with respect to the 5mC/5hmC patterns observed (Fig. 7e, f). This suggests that the 5mC/5hmC patterns of these TF genes are responsive to the environment and may undergo changes in response to different conditions. The representative biological process associated with environment-responsive TFs are shown for Glial-M7 (Fig. 7e) and Neural-M28 (Fig. 7f); green indicates loss of 5hmC and gain of 5mC under Mac-CM, whereas blue indicates gain of 5hmC and loss of 5mC under Mac-CM. Changes in 5mC and 5hmC detection between DM and Mac-CM should occur by active methylation/ demethylation activity that could be associated directly with changes in *TET* and *DNMT*, consistent with the observed changes in *TET2* and *DNMT3A* expression (Fig. 7c, d, Supplementary Fig.S6) in our study. These data suggest that these TFs could be important targets associated with more accessible chromatin regions affected by the inflammatory environment. These results may also suggest that *TET* and *DNMT* are tightly linked in regulating TFs between neuronal and non-neuronal lineage commitment (Fig. 7e, f).

In sum, these results support the hypothesis the balance between methylation and hydroxymethylation in the genome for identified TFs may be connected to hES-NSC lineage selection pathways. Notably, these data are consistent with an inflammatory environment (e.g., Mac-CM) driving a direct alteration of *TET2* expression during differentiation, and thereby potentially affecting differential methylation/hydroxymethylation balance in the genome. Recent studies of CNS development have provided insights into gene status as active vs. repressed, or even “poised’, via the combination of 5mC/5hmC marks in each genome along the differentiation process^37,41–43^. Thus, differentially modulated levels of 5mC/5hmC in genes/TFs influenced by environmental factors, like inflammation^25^, could be targets for intervention to avoid derailing hES-NSC from ‘normal’ lineage commitment that could support repair after CNS injury or disease.

### *TET2* expression increased in Neural-M28 in the in vivo injured microenvironment

Our data suggest that Glial-M7 and Neural-M28 are primed towards different lineage propensities and responses to the microenvironment. We investigated whether these differences were retained in an in vivo transplantation model, in which cells are exposed to a more complex array of cellular, molecular, and substrate environmental cues.

We used a mouse SCI model to investigate whether differences between Glial-M7 vs. Neural-M28 in vitro were sustained after transplantation in the injured CNS microenvironment, in which inflammation represents a critical set of differentiation cues (Supplementary Fig.S7)^44^. We transplanted either Glial-M7 or Neural-M28 engineered to express GFP under the safe harbor adeno-associated virus integration site 1 (AAVS-1) on human chromosome 19 (Supplementary Fig.S8). We first tested the efficiency of human cell retrieval using Fluorescence-activated cell sorting (FACS) for GFP-reporter cells, seeking to establish the longest engraftment time from which we could efficiently recover cells. Stereological engraftment data from our lab in this paradigm have shown that early cell survival and proliferation are limited, making retrieval of engrafted cells challenging^45^. However, our previous histological data also suggests that a minimum of 4-weeks engraftment time in vivo is necessary to yield sufficient time for differentiation of transplanted cells to be revealed. Accordingly, we pooled 4 animals per replicate to ensure sufficient FACS harvest of human cells for making libraries and analysis. FACS retrieval of human cells expressing the GFP-reporter at 28 days post-transplantation (dpt) yielded 13–14% cell recovery per biological replicate (Supplementary Fig.S9), which was equivalent between Glial-M7 and Neural-M28. This is not surprising, as differentiation of engrafted CNS cells resulting in extensive and elongated processes within the neuropil can be expected to dramatically impair the potential to recapture these cells by FACS^45^. Accordingly, re-isolated human cells most likely represent a population that has undergone early lineage selection but not yet begun to integrate into the host CNS. Despite this limitation, we were able to extract both RNA and genomic DNA from re-isolated engrafted Glial-M7 and Neural-M28. However, RRBS libraries failed to yield a sufficient number of mappable CpGs, likely because of a genomic DNA recovery threshold, as RNA is several-fold more abundant than DNA in a cell. Because comprehensive DML analysis was therefore not possible, we focused instead on analysis of differentially expressed genes (DEGs) by RNA-seq.

**Fig. 8:**
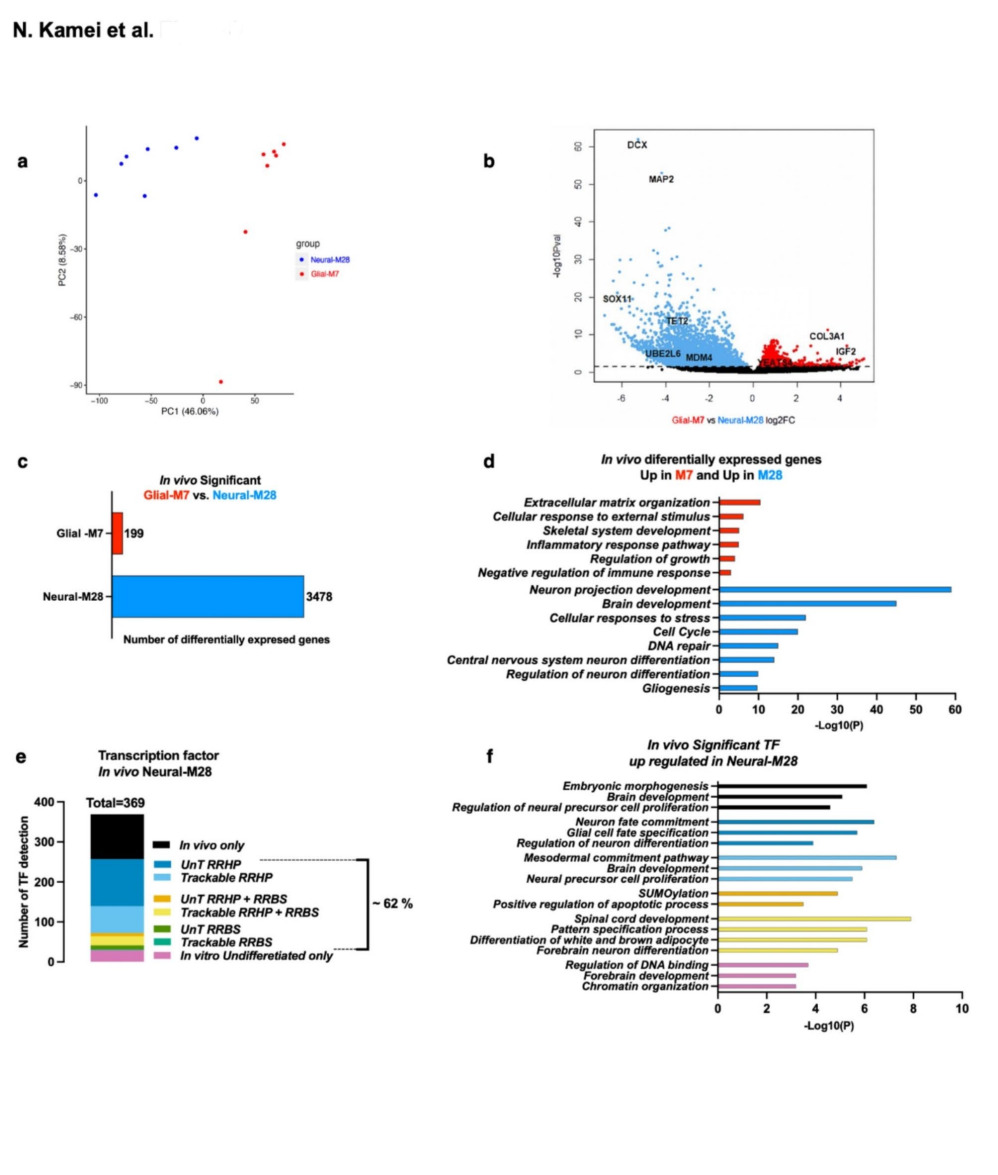
*TET2* was up-regulated under in vivo injured inflammatory environment: 9 dpi- 28 dpt paradigm in hES-NSC compared to in vitro inflammatory condition, Mac-CM. Differential transcriptome analysis from 9 days post-injury (dpi) and 28 days post-transplantation (dpt) of in vivo re-isolated engrafted Glial-M7 and Neural-M28 cells from injured NSG mouse spinal cord is shown. (**a**) The principal component analysis results between biological replicates of re-isolated engrafted Glial-M7 and Neural-M28 from RNA-seq are shown. Seven biological replicates for Glial-M7 and Neural-M28 were analyzed (see materials and methods). (**b**) shows the Volcano plot of differentially expressed genes (DEG) between Glial-M7 (Red) and Neural-M28 (Blue). (**c**) The graph shows the significantly differentially expressed genes (DEGs) from (**b)**: 199 genes are up-regulated in Glial-M7 (Red) but downregulated in Neural-M28, and 3478 genes showed decreased expression in Glial-M7 but are up-regulated in Neural-M28 (Blue). **(d)** Representative biological processes for these up-regulated in Glial-M7 or Neural-M28 are shown. (**e**) The graph shows TFs from in vivo significant DEG up-regulated in Neural-M28 compared with TFs detected in vitro by 5mC/5hmC pattern; trackable (T: pattern 8–28) or untrackable (UnT: patten 29–31, or pattern 1–7; in vitro undifferentiated stage only), which is shown in Fig. 6e, f. 62% of TFs (228 TFs) from engrafted Neural-M28 significant DEG were also detected during in vitro differentiation. **(f)** shows representative biological processes related to the TFs from graph (**e**) for Neural-M28.

Principal component analysis and a volcano plot of DEGs from the transcriptomes of recovered engrafted Glial-M7 vs. Neural-M28 cells is shown in Fig. 8a, b, & Supplementary Fig.S10, revealing stark differences between these groups. A total of 3,677 DEGs were detected as significant and identified in both Glial-M7 and Neural-M28 (Fig. 8c). Of these, 199 DEGs were up-regulated in Glial-M7 but decreased in Neural-M28, while 3,478 DEGs were decreased in Glial-M7 but up-regulated in Neural-M28. Upregulation of a larger set of DEGs in Neural-M28 vs. Glial-M7 is consistent with our in vitro observations of lineage-related gene regulation in these cells in response to differentiation in the presence of inflammatory cues. Interestingly, differential analysis of *TET2* expression identified an 11-fold increase in engrafted Neural M28 vs. Glial M7 (Fig. 8b). Given that exposure to Mac-CM during differentiation blocked upregulation of *TET2* expression in Neural M28 cells (Fig. 7d), these data suggest that the in vivo microenvironment at the time of re-isolation is associated with strong *TET2* expression.

Representative biological processes for these significant DEGs are shown in Fig. 8d. The 199 DEG upregulated in Glial-M7 re-isolated human cells represented mainly genes involved in the ECM and immune response/cellular response to external stimululi, including the hallmark genes for epithelial-mesenchymal transition (EMT), which have been reported after extended exposure to an in vivo inflammatory environment^46^. Approximately 20% were also related to regulating the growth or the immune/defense response. Only one TF gene (*YEATS4*), known as a chromatin reader component of histone acetyltransferase complex, was up-regulated in engrafted Glial-M7.

For the 3,478 DEG up-regulated in Neural-M28 re-isolated human cells, 369 represented TFs from genes related to neuronal pathways (Fig. 4b). In particular, *DCX* and *MAP2*, which are known to detect developing neurons, were highly expressed in Neural-M28. In addition, many zinc finger (ZNF) TF genes were detected; this observation could reflect the known role of ZNF genes in regulating the immune response^47^.

Finally, we also probed the overlap between in vitro and in vivo observations of identified TFs. TF detection during in vitro differentiation (Fig. 6e, f, Supplementary Table S2,3) was completed by analysis of epigenetic patterns, in contrast, analysis of re-isolated human cells was limited to RNA-seq. Despite this experimental limitation, 62% of TFs (228 TFs) from engrafted Neural-M28 significant DEG were also detected during in vitro differentiation, identifying a strong overlap (Fig. 8e, f vs. Figure 7b). However, more than half of these TF genes are only detected under normal differentiation (DM) culture conditions. These TFs potentially contribute to differentiation along the glial lineage. Additionally, 38% of the TF genes were only expressed upon in vivo transplantation of the cells. From a transcriptome view, engrafted Neural-M28 in vivo may differentiate into neurons; however, they may also be underway expansion/proliferation as neural precursor cells (Fig. 8f). These results allow us to hypothesize that cellular, molecular, or substrate factors in the in vivo microenvironment may influence Neural-M28 to promote proliferation, potentially linked to *TET2* up-regulation (Fig. 8b).

## Discussion

Up-regulation of *TET2* is a potential pluripotent exit signal that regulates the epigenetic landscape in human PSC-derived neural stem cells^48^. Our data suggest that undifferentiated hES-NSC cells have already been primed for specific gene expression (Fig. 2, Supplementary Fig.S1)^49^. We selected two cell line derivatives (Glial-M7, Neural-M28) that were derived from the same PSC origin but exhibited priming towards a glial or neuronal lineage at the undifferentiated stage. Critically, we show that an inflammatory environment (Mac-CM) differentially modulated cell lineage markers in these lines, and even overrode the effect of *TET2*/*DNMT3A* knockdown in hES-NSC. Based on these data, we analyzed genome-wide 5mC/5hmC in Glial-M7 and Neural-M28.

70% of 5mC and 5hmC detected as significant in the genome in undifferentiated Glial-M7 or Neural-M28 hES-NSC did not overlap with gene expression results by RNA-seq (Fig. 6a, b). RNA-seq results show the transcriptome at a given time for each hES-NSC, but 5mC/5hmC detection on the genome may show the holding state of what hES-NSC will become. We, therefore, hypothesized that these genes/TFs detected with only 5mC/5hmC epigenetic marks could be epigenetically primed regions that may predict their cell fate determination pathway (Fig. 6e, f)^4^. Genome-wide 5mC/5hmC analysis for Glial-M7 and Neural-M28 enabled us to track TFs for 5mC/5hmC detection through differentiation. Importantly, differentially changed detected levels of 5mC/5hmC allowed us to distinguish TFs affected by media condition, DM, or Mac-CM in each derivative. We found that *TET2* expression in Glial-M7 and Neural-M28 was down-regulated by culture in Mac-CM, an in vitro model of inflammatory conditions (Fig. 7c, d) and that this downregulation was correlated to a significant reduction in the number of TFs detected in vitro, especially for 5hmC under Mac-CM (Fig. 7a, b). Thus, Mac-CM may direct each hES-NSC towards a more discrete differentiation pathway in vitro.

The analysis of the 5mC/5hmC patterns in cells cultured under DM or Mac-CM conditions also identified the same TF genes that were associated with the same lineage pathways. However, these TF genes exhibited different 5mC/5hmC levels between the two conditions. These 5mC/5hmC changes may be triggered by the Glial-M7 or Neural-M28 *TET2* expression level during differentiation. These TFs and other genes could be important targets that may be associated with more accessible chromatin regions during the differentiation, thus, responding under different environments. Our in vitro results may support that the expression level of *TET2* influences the 5mC/5hmC balance in NSC lineage commitment (Supplementary Fig.S11). In all, these data are consistent with the previously identified importance of the *TET* genes in neural cells^8,24^ and may suggest a role for both the baseline epigenetic state and 5mC/5hmC balance in the response of NSC to their environment – in this case, inflammatory factor signaling presented by Mac-CM.

Interestingly, under an in vivo injured environment, *TET2* expression was significantly up-regulated in engrafted Neural-M28 vs. Glial-M7 (Fig. 8b). The in vivo environment of the injured spinal cord, containing a more complex array of resident CNS and responding immune cells, as well as their secreted molecular cues and an altered substrate array, may play a role in *TET2* expression level change, however, further study is needed to understand this process^50^. The in vivo three-dimensional (3D) environment is known to promote neural induction compared to 2D^51^ and the 3D environment for hNSC has shown more efficient differentiation than 2D^52^. Thus, the in vivo environment may enhance neural lineage commitment for Neural-M28, resulting in a relative elevation of *TET2* expression. Glial cells are a crucial regulator of neuroinflammation^53,54^; thus, Glial-M7 may react more strongly to a local immune response in vivo than in vitro Mac-CM, reducing the expression of genes (Fig. 8c vs. 7a).

In sum, this study showed that inflammatory environments influence the initial hES-NSC epigenetic landscape in vitro and in vivo by modulating the *TET2* expression level. Such changes may affect cell fate. Thus, these results support the hypothesis that an epigenetic modifier gene, *TET2*, is a key regulator for each hES-NSC initial epigenetic landscape and a key indicator of inflammatory environments to which hES-NSCs may be exposed. The resulting difference in the epigenetic landscape with different initial lineage priming, could be informative to predict differences for in vivo engraftment and differentiation when these same cells are transplanted into the naïve versus injured brain or spinal cord^45,55,56^. In this regard, the identification of differential 5mC/5hmC levels at TF genes allowed us to identify factors that are influenced by the environment to which the cells were exposed. Dissection and understanding of 5mC/5hmC patterns may allow detection of TF/gene targets restricted by environmental factors to control cell fate.

## Methods

### Ethics statements

All animal housing conditions, surgical procedures, and postoperative cares are approved by the University of California, Irvine (UCI) Institutional Animal Care and Use Committee (IACUC). All animal experiments in this study were performed in accordance with ARRIVE guidelines. Usage of human neural stem cell lines Glial-M7 and Neural-M28 (derived from the same Shef6 hESC parent/origin line) and their GFP-reporter lines for all in vitro and in vivo work were reviewed and approved by the University of California, Irvine (UCI) Human Stem Cell Research Oversight Committee (hSCRO). We confirm that all methods in this study were conducted according to the relevant guidelines and regulations of IACUC and hSCRO at UCI.

### Human neural stem cell lines, culture and differentiation

Cell lines used in this study: Shef4 and Shef6 ESC^15^ (UKSCB; R-05-029, R-05-031/NIHhESC-10-0078)), H9 (WA09;WiCell), iPSC6-9 (DF6-9-9 TB; WiCell), iPSC19-9(DF19-9-11; WiCell). All hNSC are created under the same protocol and media with Xeno-free conditions^15^. Glial-M7, Neural-M7, and M28 derived from human Shef6 ESC and Glial-M7 and Neural-M28 GFP-reporter lines are established in UCI^14,15^ following all appropriate hSCRO and IBC protocols. The iPS cell line ADRC iPS-1 and NSCs derived from this line were provided by the University of California Irvine Alzheimer’s Disease Research Center (UCI-ADRC).

### In vitro differentiation under normal vs. inflammation

We cultured undifferentiated hES-NSC in Neural stem media. We dissociated them into single cells, then re-plated them with a certain number of cells that we optimized and allowed them to differentiate in the plate for 14 days. These dissociated cells are re-plated as undifferentiated cells for 24 h to attach, where we change the media to differentiation media, either normal neural differentiation media (DM) or Macrophage-conditioned media (Mac-CM). Mac-CM was generated from a pure population of macrophages isolated from peritoneal cavities of adult Rats after sodium caseinate injection. The method enables the generation of a pure population of macrophages by virtue of the timing and the isolation of sufficient cells to generate Mac-CM as described before^43^. As described above, we can isolate samples and detect results from each biologial replicate before and after treatment with two different differentiating media, DM or Mac-CM, to compare in vitro differentiation experiments in this study.

### Quantitative PCR (qPCR) for selected gene expressions

Total RNA was prepared with DNA-RNA shields followed by Quick RNA mini prep (Zymo Research (ZR)). Removed genomic DNA and created cDNA and performed qPCR on ViiA7^™^ (Thermo Fisher Scientific) followed by relative quantification using the ΔΔCt method. Taqman^®^ custom gene expression assays (Thermo Fisher Scientific), other primer sets used in this study are shown in Supplementary Table S1. All data from qPCR in this study were performed in separate biological triplicates with technical triplicates of each biological replicate. Error bar represents the standard error of the mean (SEM; *n* = 3).

### Immunocytochemistry

The method used in this study was described before^15^.

### *TET2* and *DNMT3A* knockdown in undifferentiated Glial-M7 and Neural-M28

Gene knockdown (KD) in Glial-M7 and Neural-M28 and with following differentiation were performed with transient Lentivirus shRNA transduction. We created Lentiviral shRNA from each shRNA construct (Genecopoeia) at UC Davis Medical Center vector core and tested KD efficiency for each shRNA. Mixture of the selected shRNA lentivirus were used for KD experiment for this study. Each shRNA was constructed as: H1 promoter-shRNA- SV40 promoter-reporter gene mCherry-IRES-Puromycin in the psi-LVRH1MP lentiviral vector. The virus that carries shRNA construct was measured by qPCR-based RNA titration kit and transduction titer was detected at UC Davis Vector Core. Three days after transduction with the mixture of lentiviral shRNA and LentiX-accelerator (Takara), differentiation was performed under the normal differentiation media (DM) or Mac-CM. All KD results were only analyzed for mCherry expressed cells sorted by FACS (BD Aria II) for undifferentiated hES-NSC, and differentiated hNSC for 14 days under DM and Mac-CM.

### Glial-M7 and Neural-M28 GFP reporter line

We created the reporter gene green fluorescence protein (GFP) under the CAG promoter^57^ into CompoZr-pZDonor-AAVS1-Puromycin vector and engineered parental hNSC of either Glial-M7 or Neural-M28 to express GFP constitutively using ZFN system (Sigma-Aldrich) by Nucleofection (Lonza). Especially for Glial-M7 was not constructed with a single population with a different growth rate compared to Neural-M28 (Supplementary Fig.S3); thus, we did not clone them by titration of GFP expression cells for the purpose of maintaining their original feature.

### Animals/spinal cord contusion injury

Under 2% isoflurane anesthesia, female NSG mice (*n* = 56; The Jackson Laboratory) received a laminectomy followed by a 50 kd contusion spinal cord injury (SCI) with the infinite Horizon Impactor at Vertebral T9^44^. After 9 days post injury (9dpi) each animal received a total of either Glial-M7-GFP or Neural-M28-GFP hES-NSC transplantation. Twenty-eight days post-transplantation (28 dpt), animals used for flow cytometric analyses of engrafted hES-NSC (four animals per group and time point) were euthanized by CO_2_ asphyxiation with the secondary measure of decapitation. Tissue from spinal segments T8-T10 of each animal was rapidly dissected (approximately 6 mm in total length of the spinal cord) for re-isolation of engrafted hNSC^45^. Post-SCI care included administration of lactated ringers (50 ml/kg, s.c.) and Buprenorphine (0.5 mg/kg) twice per day beginning immediately after surgery and continuing for 48 h thereafter. Mice also received antibiotic (Baytril, 2.5 mg/kg s.c.) beginning immediately after surgery and continuing daily for 5 days thereafter. We employed constitutively immunodeficient NSG mice, which lack T and B cells but have innate immune cells, and no immunosuppressant drugs were administered. To assess the consistency of SCI across animals, locomotor function was assessed in all animals using the Basso Mouse Scale^58^; BMS testing was conducted by observers blinded to study groups at 4 dpi and 27 dpt. NSG mice are immunodeficient but have an intact innate immune response.

### Preparation, transplantation, and re-isolation of engrafted cells

Glial-M7-GFP and Neural-M28-GFP hES-NSC were cultured under the normal growth media and prepared for transplantation on the day of transplantation. Cells were dissociated and re-suspended to X-Vivo 15 media, and each animal received four injections of either Glial-M7 or Neural-M28 for a total of 2 × 10^5^ cells in the intact parenchyma adjacent to the T9 contusion site (two sites at the rostral, two sites at the caudal to the injury epicenter)^59^. SC that was dissected from each animal at 28 dpt was dissociated with 1.5 mg/ml Collagenase-HBSS buffer followed by TrypLE (Fisher Scientific) treatment. Dissociated cells are passed through a 40-µm filter and sorted as the engrafted cells by GFP expression using FACS (BD Aria II) directly into 2x DNA-RNA shields (ZR).

Each transplantation group (Glial-M7-GFP or Neural-M28-GFP) has been prepared for seven biological replicates and DNA and RNA of one sample are derived from four animals’ SC. DNA and RNA from FAC-sorted cells were prepared by the Duet DNA/RNA kit (ZR) according to the manufacturer’s recommendation. The quality and quantity of DNA and RNA from the same sample were analyzed on Qubit/Agilent Bioanalyzer 2100 (Agilent).

### Samples for RNA-seq, RRBS, and RRHP

Two biological replicates for each cell line, Glial-M7, Neural-M7, and Neural-M28, were cultured as described. The undifferentiated cells were re-plated and replaced with either normal differentiation media (DM) or inflammatory conditioned media (Mac-CM) for 14 days. For in vitro samples, total RNA and genomic DNA were isolated for each biological replicate at each stage and condition and prepared for further analyses. All coordinates are on the hg38 genome build.

### RNA-seq library Preparation

RNA libraries were prepared from undifferentiated hES-NSC (in vitro), and re-isolated engrafted hES-NSC from in vivo SCI animals at 28 days post-transplantation by FAC-sorting by their GFP expression were prepared with both 10 ng of total RNA using SMARTer stranded Total RNA-seq-Pico kit (TAKARA) following the manufacturer’s protocol.

### Methyl-MiniSeq™ library Preparation (RRBS)

Genome-wide DNA methylation profiling using RRBS-style enzymatic enrichment has been extensively described elsewhere^25,26^. Here, libraries were prepared from 100 ng genomic DNA using two restriction enzymes (Msp I and Taq alpha I) that recognize CG-dense fractions of the genome to double the number of profiled CpG sites compared to standard protocols.

### Hydroxymethyl Seq™ Library Preparation (RRHP)

Libraries were prepared according to the method of Petterson, et al.^27^. Briefly, 5hmC modified cytosine was labeled with a glucose moiety that leaves them alternately recognizable to restriction endonuclease digestion. Protected DNA fragments are enriched during NGS library preparation and make up the vast majority of reads after sequencing.

### RNA-seq data processing and analysis

RNA-seq reads for all data were processed and checked by FastQC. Duplication % was ~ 60% that were commonly detected on human RNA-seq, and overall alignment rates were > 95% for all samples. Data were analyzed using RseQC v4.0.0 (http://rseqc.sourceforge.net/*)* and validated. We then performed Principal component analysis (PCA) and created the heatmap using Log2 of FPKM. Differentially expressed genes were detected by the DEseq2 program^60^. The significance was defined as adjusted p-value < 0.05 and at least a 2-fold change in either direction.

### Methyl-MiniSeq™ sequence alignments and data analysis

Sequence reads from bisulfite-treated libraries were identified using standard Illumina base-calling software and Bismark (http://www.bioinformatics.babraham.ac.uk/projects/bismark/*)* to perform the alignment. Index files were constructed using the Bismark_genome_preparation command and the entire reference genome. The non-directional parameter was applied while running Bismark. All other parameters were set to default. Filled-in nucleotides were trimmed off when methylation calling was done. The DSS (Dispersion Shrinkage for Sequencing data) program^28^ is designed for differential analysis of high-throughput sequencing data for processed bisulfite sequence results to detect differential methylation loci (DML). We also used thresholding of adjusted p-value < 0.05 and at least a 10% change in methylation between groups in all pairwise comparisons cased for filtering. Annotation calling for each CpG site was determined using the Goldmine package^61^. We also confirmed that each CpG called as a DML in at least one of the pairwise comparisons done by DSS showed that the DML called were robust.

### Hydroxymethyl Seq™ sequence alignments and data analysis

Sequence reads from RRHP libraries were first processed to trim off the low-quality bases and the P7CG adapter at the 3’ end of the reads. Reads were then aligned to the reference genome using the Bowtie default parameters. Aligned reads with the Msp I tag (CCGG) were counted. The correlation analysis between different RRHP libraries was performed by comparing the presence of the tagged reads at each profiled Msp I site, and Pearson’s coefficient was calculated accordingly. DHML was analyzed by DEseq2 and detected 32,602 CCGG genes that were detected for each strand separately and are significant with an adjusted p-value < 0.05 of at least a 2-fold change in either direction for each pairwise comparison. Each pairwise comparison: Log2FoldChange, adjusted p-value (padj) for the comparison and add significance with the combination of the padj < 0.05 and Log2 Fold change > 1.

### Principal component analysis (PCA) and heatmap

PCA analysis was performed by using the prcomp function of stats v3.6.2 (https://www.rdocumentation.org/packages/stats/versions/3.6.2/topics/prcomp ) for Fig. 5. The heatmaps of RNA seq, DNA methylation, and DNA hydroxymethylation were generated with heatmap.2 function of gplots v3.1.1 in R v3.0 (https://cran.r-project.org/web/packages/gplots/index.html*).*

### Annotated gene analysis

Annotated sites (DML/DHML) detected from differential analysis of DMS and DHMS, explained above, were identified using the Goldmine R package (http://www.jeffbio.com/goldmine/) ^61^. By extending each DML by +/- 2 kb and merged all of those extended regions sustain changes detected from the initial differential analysis^61^. Gene ontology analysis was performed using Metascape^62^.

## Supplementary Information

Supplementary Infomation 1.

Supplementary Infomation 2.

Supplementary Infomation 3.

## Data Availability

Data supporting the results reported in the article can be found in the Bioproject repository (https://www.ncbi.nlm.nih.gov/bioproject/) under accession ID PRJNA1027314.

## References

[1] Lister, R. et al. Global Epigenomic Reconfiguration During Mammalian Brain Development. *Science (80).* 341, (2013). 10.1126/science.1237905 PMC3785061 23828890

[2] Stein, J. L. et al. A quantitative Framework to evaluate modeling of cortical development by neural stem cells. *Neuron*. **83**, 69–86 (2014). 24991955 10.1016/j.neuron.2014.05.035 PMC4277209

[3] Kriaucionis, S. & Heintz, N. The nuclear DNA base 5-Hydroxymethylcytosine is Present in Purkinje neurons and the brain. *Sci. (80-)*. **324**, 929–930 (2009). 10.1126/science.1169786 PMC3263819 19372393

[4] Hon, G. C. et al. 5mC oxidation by Tet2 modulates enhancer activity and timing of Transcriptome Reprogramming during differentiation. *Mol. Cell. ***56**, 286–297 (2014). 25263596 10.1016/j.molcel.2014.08.026 PMC4319980

[5] Li, X. et al. Tet proteins influence the balance between neuroectodermal and mesodermal fate choice by inhibiting Wnt signaling. *Proc. Natl. Acad. Sci.* 113, E8267 LP-E8276 (2016). 10.1073/pnas.1617802113 PMC5187696 27930333

[6] Wu, X., Li, G. & Xie, R. Decoding the role of TET family dioxygenases in lineage specification. *Epigenetics Chromatin*. **11**, 58 (2018). 30290828 10.1186/s13072-018-0228-7 PMC6172806

[7] Szulwach, K. E. et al. 5-hmC-mediated epigenetic dynamics during postnatal neurodevelopment and aging. *Nat. Neurosci. ***14**, 1607–1616 (2011). 22037496 10.1038/nn.2959 PMC3292193

[8] Hahn, M. A. et al. Dynamics of 5-Hydroxymethylcytosine and chromatin marks in mammalian neurogenesis. *Cell. Rep. ***3**, 291–300 (2013). 23403289 10.1016/j.celrep.2013.01.011 PMC3582786

[9] Wang, T. et al. Genome-wide DNA hydroxymethylation changes are associated with neurodevelopmental genes in the developing human cerebellum. *Hum. Mol. Genet. ***21**, 5500–5510 (2012). 23042784 10.1093/hmg/dds394 PMC3516134

[10] Bachman, M. et al. 5-Hydroxymethylcytosine is a predominantly stable DNA modification. *Nat. Chem. ***6**, 1049 (2014). 25411882 10.1038/nchem.2064 PMC4382525

[11] Mellén, M., Ayata, P. & Heintz, N. 5-hydroxymethylcytosine accumulation in postmitotic neurons results in functional demethylation of expressed genes. *Proc. Natl. Acad. Sci.* 114, E7812 LP-E7821 (2017). 10.1073/pnas.1708044114 PMC5604027 28847947

[12] Szulwach, K. E. et al. Integrating 5-Hydroxymethylcytosine into the Epigenomic Landscape of Human Embryonic Stem cells. *PLoS Genet. ***7**, e1002154 (2011). 21731508 10.1371/journal.pgen.1002154 PMC3121778

[13] Ziller, M. J. et al. Dissecting neural differentiation regulatory networks through epigenetic footprinting. *Nature*. **518**, 355–359 (2015). 25533951 10.1038/nature13990 PMC4336237

[14] Nguyen, H. X. et al. Induction of early neural precursors and derivation of tripotent neural stem cells from human pluripotent stem cells under xeno-free conditions. *J. Comp. Neurol. ***522**, 2767–2783 (2014). 24715528 10.1002/cne.23604

[15] Haus, D. L. et al. CD133-enriched Xeno-Free human embryonic-derived neural stem cells expand rapidly in culture and do not form teratomas in immunodeficient mice. *Stem Cell. Res. ***13**, 214–226 (2014). 25082219 10.1016/j.scr.2014.06.008 PMC5675021

[16] Kriegstein, A. & Alvarez-Buylla, A. The glial nature of embryonic and adult neural stem cells. *Annu. Rev. Neurosci. ***32**, 149–184 (2009). 19555289 10.1146/annurev.neuro.051508.135600 PMC3086722

[17] Ebert, A. D. et al. EZ spheres: a stable and expandable culture system for the generation of pre-rosette multipotent stem cells from human ESCs and iPSCs. *Stem Cell. Res. ***10**, 417–427 (2013). 23474892 10.1016/j.scr.2013.01.009 PMC3786426

[18] Wang, H. et al. Role of CD133 in human embryonic stem cell proliferation and teratoma formation. *Stem Cell. Res. Ther. ***11**, 208 (2020). 32460847 10.1186/s13287-020-01729-0 PMC7251672

[19] Wu, Z. et al. Dnmt3a regulates both proliferation and differentiation of mouse neural stem cells. *J. Neurosci. Res. ***90**, 1883–1891 (2012). 22714992 10.1002/jnr.23077 PMC3418436

[20] Zhang, X. et al. DNMT3A and TET2 compete and cooperate to repress lineage-specific transcription factors in hematopoietic stem cells. *Nat. Genet. ***48**, 1014–1023 (2016). 27428748 10.1038/ng.3610 PMC4957136

[21] Beck, K. D. et al. Quantitative analysis of cellular inflammation after traumatic spinal cord injury: evidence for a multiphasic inflammatory response in the acute to chronic environment. *Brain*. **133**, 433–447 (2010). 20085927 10.1093/brain/awp322 PMC2858013

[22] Wu, H. et al. Dnmt3a-Dependent nonpromoter DNA methylation facilitates transcription of neurogenic genes. *Sci. (80-)*. **329**, 444–448 (2010). 10.1126/science.1190485 PMC3539760 20651149

[23] He, Y. & Ecker, J. R. Non-CG methylation in the Human Genome. *Annu. Rev. Genomics Hum. Genet. ***16**, 55–77 (2015). 26077819 10.1146/annurev-genom-090413-025437 PMC4729449

[24] MacArthur, I. C. & Dawlaty, M. M. TET enzymes and 5-Hydroxymethylcytosine in neural Progenitor Cell Biology and Neurodevelopment. *Front. cell. Dev. Biol. ***9**, 645335 (2021). 33681230 10.3389/fcell.2021.645335 PMC7930563

[25] Jiang, S. et al. Intra-individual methylomics detects the impact of early-life adversity. *Life Sci. Alliance*. **2**, e201800204 (2019). 30936186 10.26508/lsa.201800204 PMC6445397

[26] Meissner, A. et al. Reduced representation bisulfite sequencing for comparative high-resolution DNA methylation analysis. *Nucleic Acids Res. ***33**, 5868–5877 (2005). 16224102 10.1093/nar/gki901 PMC1258174

[27] Petterson, A., Chung, T. H., Tan, D., Sun, X. & Jia, X. Y. RRHP: a tag-based approach for 5-hydroxymethylcytosine mapping at single-site resolution. *Genome Biol. ***15**, 456 (2014). 25248841 10.1186/s13059-014-0456-5 PMC4212096

[28] Feng, H., Conneely, K. N. & Wu, H. A bayesian hierarchical model to detect differentially methylated loci from single nucleotide resolution sequencing data. *Nucleic Acids Res. ***42**, e69–e69 (2014). 24561809 10.1093/nar/gku154 PMC4005660

[29] Anders, S. & Huber, W. Differential expression analysis for sequence count data. *Genome Biol. ***11**, R106 (2010). 20979621 10.1186/gb-2010-11-10-r106 PMC3218662

[30] Tsankov, A. M. et al. Transcription factor binding dynamics during human ES cell differentiation. *Nature*. **518**, 344–349 (2015). 25693565 10.1038/nature14233 PMC4499331

[31] Lee, H. K., Lee, H. S. & Moody, S. A. Neural transcription factors: from embryos to neural stem cells. *Mol. Cells*. **37**, 705–712 (2014). 25234468 10.14348/molcells.2014.0227 PMC4213760

[32] Niwa, H. The principles that govern transcription factor network functions in stem cells. *Development*. **145**, dev157420 (2018). 29540464 10.1242/dev.157420

[33] Leeb, M. & Wutz, A. Establishment of epigenetic patterns in development. *Chromosoma*. **121**, 251–262 (2012). 22427185 10.1007/s00412-012-0365-x PMC3350763

[34] Mellen, M., Ayata, P., Dewell, S., Kriaucionis, S. & Heintz, N. MeCP2 binds to 5hmC enriched within active genes and accessible chromatin in the nervous system. *Cell*. **151**, 1417–1430 (2012). 23260135 10.1016/j.cell.2012.11.022 PMC3653293

[35] Luo, C., Hajkova, P. & Ecker, J. R. Dynamic DNA methylation: In the right place at the right time. *Science (80-.).* 361, 1336 LP – 1340 (2018). 10.1126/science.aat6806 PMC6197482 30262495

[36] Matsumura, Y. et al. H3K4/H3K9me3 bivalent chromatin domains targeted by lineage-specific DNA methylation pauses adipocyte differentiation. *Mol. Cell. ***60**, 584–596 (2015). 26590716 10.1016/j.molcel.2015.10.025

[37] Shi, D. Q., Ali, I., Tang, J. & Yang, W. C. New insights into 5hmC DNA modification: generation, distribution and function. *Front. Genet. ***8**, 100 (2017). 28769976 10.3389/fgene.2017.00100 PMC5515870

[38] Yu, M. et al. Base-resolution analysis of 5-Hydroxymethylcytosine in the mammalian genome. *Cell*. **149**, 1368–1380 (2012). 22608086 10.1016/j.cell.2012.04.027 PMC3589129

[39] Hu, L. et al. Crystal structure of TET2-DNA complex: insight into TET-Mediated 5mC oxidation. *Cell*. **155**, 1545–1555 (2013). 24315485 10.1016/j.cell.2013.11.020

[40] Hodges, E. et al. Directional DNA methylation changes and Complex Intermediate States Accompany Lineage specificity in the adult hematopoietic compartment. *Mol. Cell. ***44**, 17–28 (2011). 21924933 10.1016/j.molcel.2011.08.026 PMC3412369

[41] Huang, Y. et al. Distinct roles of the methylcytosine oxidases Tet1 and Tet2 in mouse embryonic stem cells. *Proc. Natl. Acad. Sci. U. S. A.* 111, 1361–1366 (2014). 10.1073/pnas.1322921111 PMC3910590 24474761

[42] Pastor, W. A. et al. Genome-wide mapping of 5-hydroxymethylcytosine in embryonic stem cells. *Nature*. **473**, 394–397 (2011). 21552279 10.1038/nature10102 PMC3124347

[43] Wagner, J. et al. The relationship between DNA methylation, genetic and expression inter-individual variation in untransformed human fibroblasts. *Genome Biol. ***15**, R37 (2014). 24555846 10.1186/gb-2014-15-2-r37 PMC4053980

[44] Hooshmand, M. J. et al. Neutrophils Induce Astroglial Differentiation and Migration of Human Neural Stem Cells via C1q and C3a Synthesis. *J. Immunol.* 199, 1069 LP – 1085 (2017). 10.4049/jimmunol.1600064 PMC5523578 28687659

[45] Sontag, C., Uchida, N., Cummings, B. & Anderson, A. Injury to the spinal cord Niche alters the Engraftment dynamics of Human neural stem cells. *Stem Cell. Rep. *10.1016/j.stemcr.2014.03.005 (2014). 10.1016/j.stemcr.2014.03.005 PMC4050489 24936450

[46] Kalluri, R. & Weinberg, R. A. The basics of epithelial-mesenchymal transition. *J. Clin. Invest. ***119**, 1420–1428 (2009). 19487818 10.1172/JCI39104 PMC2689101

[47] Rakhra, G. & Rakhra, G. Zinc finger proteins: insights into the transcriptional and post transcriptional regulation of immune response. *Mol. Biol. Rep. ***48**, 5735–5743 (2021). 34304391 10.1007/s11033-021-06556-x PMC8310398

[48] Bagci, H. & Fisher, A. G. DNA demethylation in pluripotency and reprogramming: the role of Tet proteins and Cell Division. *Cell. Stem Cell. ***13**, 265–269 (2013). 24012367 10.1016/j.stem.2013.08.005

[49] Kong, L. et al. A primary role of TET proteins in establishment and maintenance of De Novo bivalency at CpG islands. *Nucleic Acids Res. ***44**, 8682–8692 (2016). 27288448 10.1093/nar/gkw529 PMC5062965

[50] Quraishe, S., Forbes, L. H. & Andrews, M. R. The Extracellular Environment of the CNS: Influence on Plasticity, Sprouting, and Axonal Regeneration after Spinal Cord Injury. *Neural Plast.* 2952386 (2018). (2018). 10.1155/2018/2952386 PMC5932463 29849554

[51] Chandrasekaran, A. et al. Comparison of 2D and 3D neural induction methods for the generation of neural progenitor cells from human induced pluripotent stem cells. *Stem Cell. Res. ***25**, 139–151 (2017). 29128818 10.1016/j.scr.2017.10.010

[52] Song, S. et al. Controlling properties of human neural progenitor cells using 2D and 3D conductive polymer scaffolds. *Sci. Rep. ***9**, 19565 (2019). 31863072 10.1038/s41598-019-56021-w PMC6925212

[53] Colombo, E., Farina, C. & Astrocytes Key regulators of Neuroinflammation. *Trends Immunol. ***37**, 608–620 (2016). 27443914 10.1016/j.it.2016.06.006

[54] Yang, Q. & Zhou, J. Neuroinflammation in the central nervous system: Symphony of glial cells. *Glia*. **67**, 1017–1035 (2019). 30548343 10.1002/glia.23571

[55] Haus, D. L. et al. Transplantation of human neural stem cells restores cognition in an immunodeficient rodent model of traumatic brain injury. *Exp. Neurol. ***281**, 1–16 (2016). 27079998 10.1016/j.expneurol.2016.04.008

[56] Cummings, B. J., Hooshmand, M. J., Salazar, D., e, L. & Anderson, A. J. Chapter fifteen. Human neural stem CellMediated Repair of the Contused spinal cord: timing the Microenvironment. *Dev. Degener Regen Nerv. Syst. ***1**, 297–323 (2008).

[57] Liew, C. G., Draper, J. S., Walsh, J., Moore, H. & Andrews, P. W. Transient and stable transgene expression in human embryonic stem cells. *Stem Cells*. **25**, 1521–1528 (2007). 17379764 10.1634/stemcells.2006-0634

[58] Basso, D. M. *et al.* Basso Mouse Scale for Locomotion Detects Differences in Recovery after Spinal Cord Injury in Five Common Mouse Strains. *J Neurotrauma* **23**, 635–659 (2006). 10.1089/neu.2006.23.635 16689667

[59] Cummings, B. J. et al. Human neural stem cells differentiate and promote locomotor recovery in spinal cord-injured mice. *Proc. Natl. Acad. Sci. U. S. A.* 102, 14069–14074 (2005). 10.1073/pnas.0507063102 PMC1216836 16172374

[60] Love, M. I., Huber, W. & Anders, S. Moderated estimation of Fold change and dispersion for RNA-seq data with DESeq2. *Genome Biol. ***15**, 550 (2014). 25516281 10.1186/s13059-014-0550-8 PMC4302049

[61] Bhasin, J. M. & Ting, A. H. Goldmine integrates information placing genomic ranges into meaningful biological contexts. *Nucleic Acids Res. ***44**, 5550–5556 (2016). 27257071 10.1093/nar/gkw477 PMC4937336

[62] Zhou, Y. et al. Metascape provides a biologist-oriented resource for the analysis of systems-level datasets. *Nat. Commun. ***10**, 1523 (2019). 30944313 10.1038/s41467-019-09234-6 PMC6447622

