# Supplementary material for: Injured inflammatory environment overrides the TET2 shaped epigenetic landscape of pluripotent stem cell derived human neural stem cells
Source: Sci Rep. 2024 Oct 24;14:25186. doi: 10.1038/s41598-024-75689-3 (PMC11502794; doi:10.1038/s41598-024-75689-3)
Supplement: Supplementary file 1 — Supplementary Infomation 1. [file 41598_2024_75689_MOESM1_ESM.pdf]

Injured Inflammatory Environment Overrides the *TET2* Shaped Epigenetic Landscape of Pluripotent Stem Cell Derived Human Neural Stem Cells

Noriko Kamei[1,3*], Kenneth Day[2,4], Wei Guo[2], Daniel L. Haus[1], Hal X. Nguyen[1], Vanessa M. Scarfone[1], Keith Booher[2], Xi-Yu Jia[2], Brian J. Cummings[1*], and Aileen J. Anderson[1*]

1 Sue & Bill Gross Stem Cell Research Center, University of California, Irvine, Irvine, CA 92697-1705, USA

2 Zymo Research Corp. 17062 Murphy Ave, Irvine, California, 92614, USA

3 Anatomy and Neurobiology, University of California, Irvine, Irvine, CA 92697-4475, USA

4 Vidium Animal Health, 7201 E Henkel Way Suite210, Scottsdale, AZ 85255, USA

* Corresponding authors

Noriko Kamei (nkamei@uci.edu), Brian J. Cummings (cummings@uci.edu), Aileen J. Anderson (aja@uci.edu)

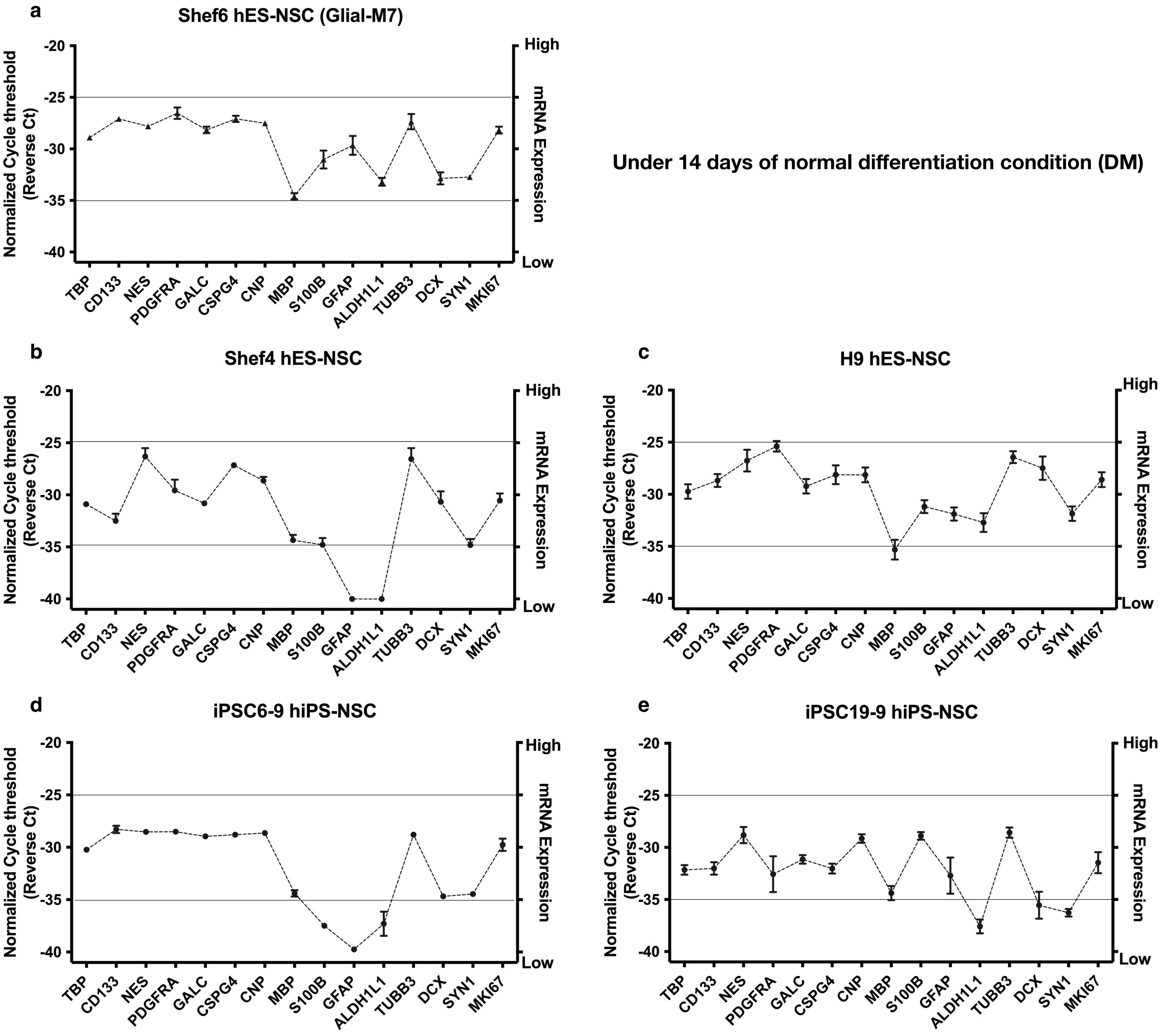

Supplementary Figure S1

Gene expression results are shown of a selected gene panel of each hES-NSC and hiPS-NSC shown in Fig. 1 under normal differentiation media (DM) for 14 days *in vitro*. The dotted line shown in each graph is only for visual purposes and does not imply direct relationships.

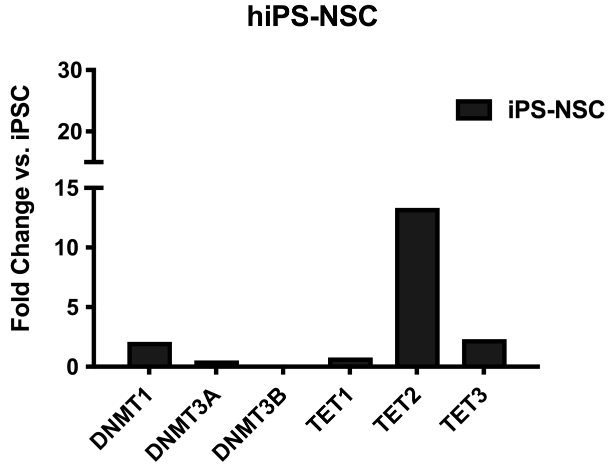

Supplementary Figure S2

***TET2* gene expression is up-regulated in iPS-NSC created by a second method.**

The fold change of DNA methyltransferase gene (*DNMT1*, *3A*, and *3B*) and Methylcytosine deoxygenase gene (*TET1*, *2*, and *3*) expressions are shown for iPS-hNSC (dark bars) compared to the original induced pluripotent stem cells (n=1). The iPS cell line ADRC iPS-1 and NSCs, derived from this line, were provided by the University of California Irvine Alzheimer's Disease Research Center (UCI-ADRC).

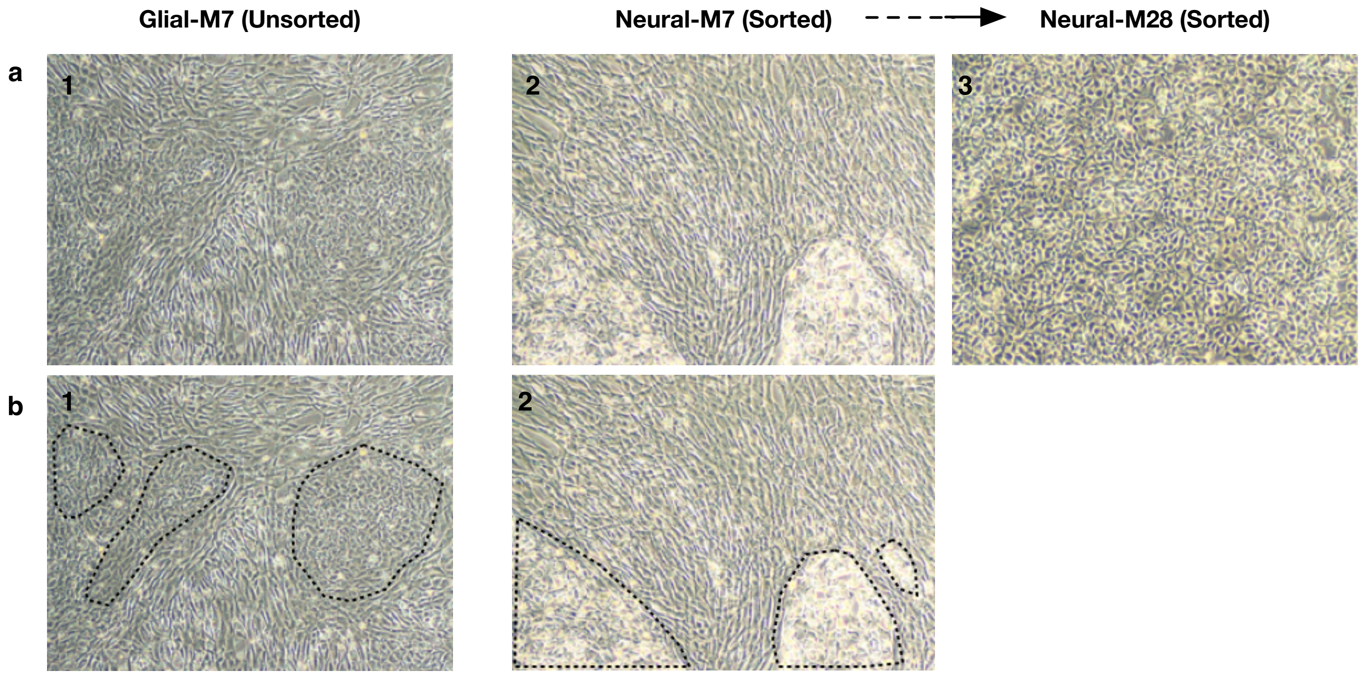

Supplementary Figure S3

Two morphologically different cell types are observed in Glial-M7, but only the smaller cell type is observed in Neural-M28.

Inverted microscopic images for Glial-M7 (Unsorted, **a1**, **b1**), Neural-M7 (Sorted, **a2**, **b2**), and Neural-M28 (Sorted, **a3**) undifferentiated hES-NSC that were used in this study are shown. Glial-M7 shows large flatter cells (majority) with clusters of smaller cells (**b1**; dotted line shows the cluster of smaller cells). Neural-M7 shows still two types of cells as Glial-M7 but the smaller cell ratio compared to the flatter cells was increased compared to Glial-M7. Also, the smaller cells grew faster than the large flatter cells, thus the clusters of smaller cells in the dot line showed slightly overgrowth stage (**b2**). Neural-M28 shows uniform small cells that grow fast.

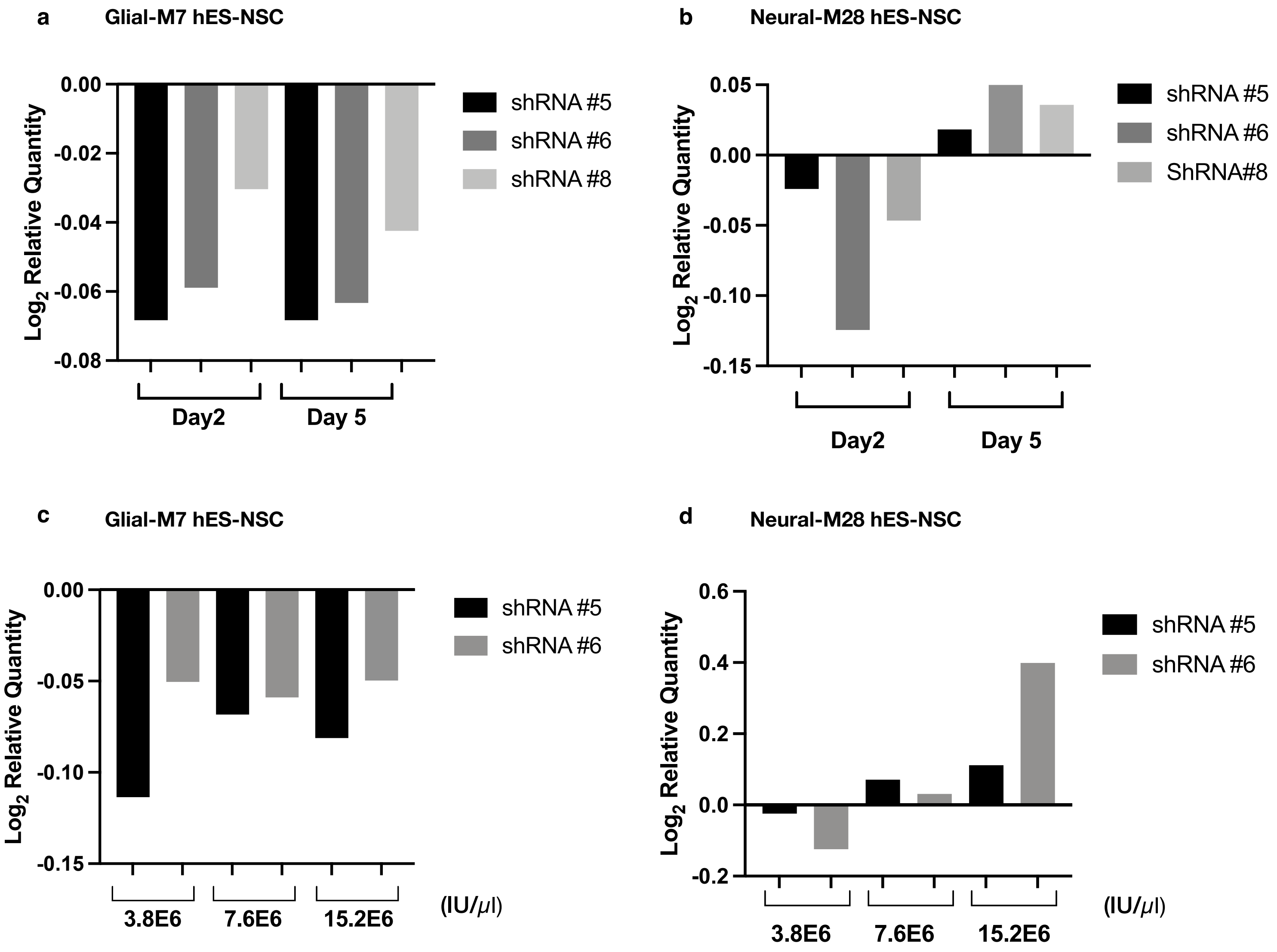

Supplementary Figure S4

***TET2* KD instability was detected in undifferentiated Neural-M28 compared to undifferentiated Glial-M7.** Example of *TET2* KD by the *TET2* shRNA constructs #5, 6, & 8 for Glial-M7 (**a, c**) and Neural-M28 (**b, d**) are shown. *TET2* knockdown level was detected at 2 days or 5 days after KD are shown in (**a**) and (**b**). The example relationships between the amount of lentivirus that carries *TET2* shRNA (see Material and Methods) used for KD experiment and *TET2* KD level are shown in (**c**) and (**d**). Lentiviral RNA was measured by qPCR based viral RNA titration kit (amb.Inc).

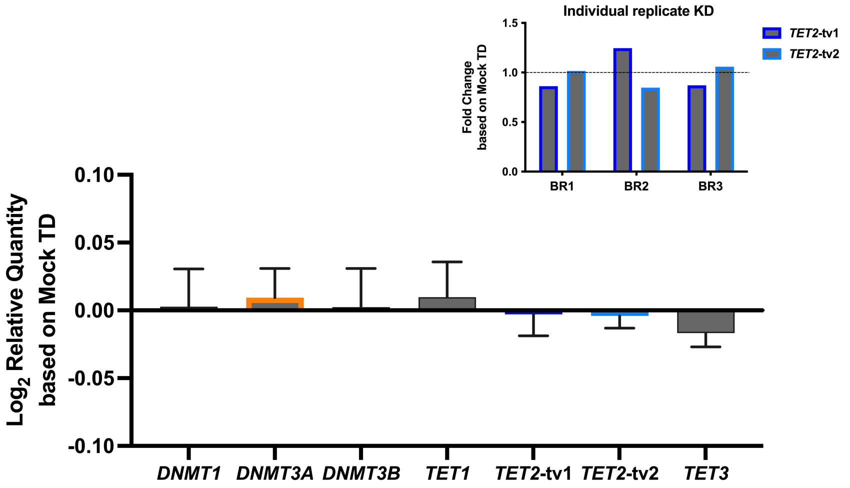

Supplementary Figure S5

***TET2* KD did not work efficiently in undifferentiated Neural-M28.**

TET2 KD was inefficient and did not show statistically significant results in undifferentiated Neural-M28. Either *TET2* tv1 or tv2 expression was maintained or increased in each biological replicate (inset graph). The results show that Neural-M28 could be resistance to *TET2* gene KD.

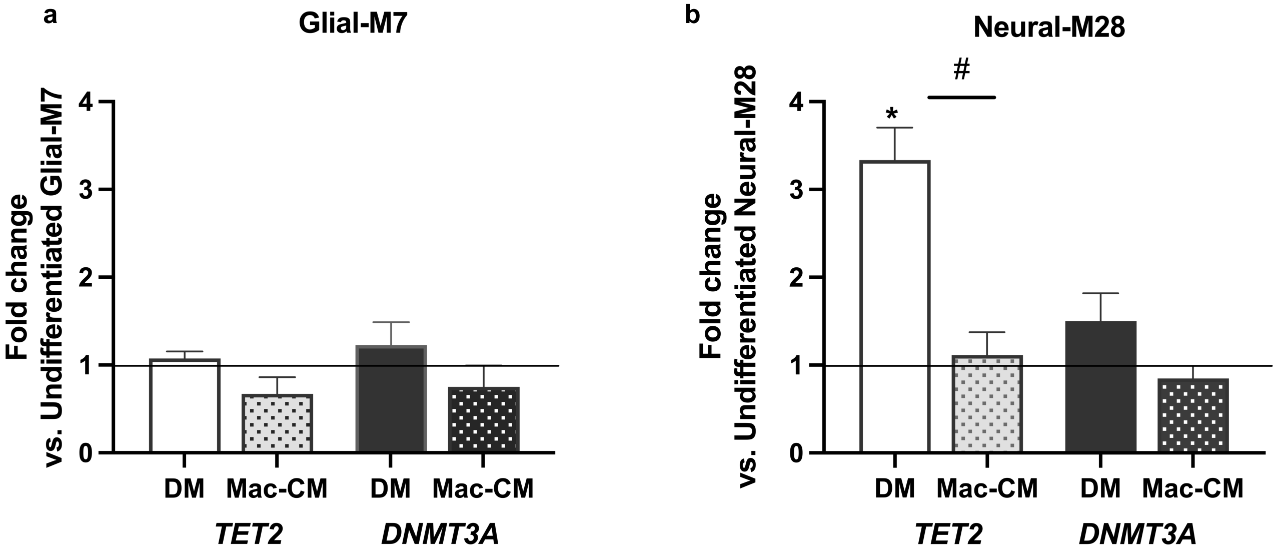

Supplementary Figure S6

***TET2* and *DNMT3A* expression levels may be linked and down regulated under macrophage conditioned differentiation media (Mac-CM) for each cell line.**

The graphs show the fold change of *TET2* and *DNMT3A* gene expression for Glial-M7 (a) and Neural-M28 (b) under Normal (DM) or macrophage-conditioned (Mac-CM) differentiation media analyzed based on the expression level of each gene in each cell line's undifferentiated hES-NSC *in vitro*. *TET2* expression level is higher than *DNMT3A* expression level for both Glial-M7 and Neural-M28 under both conditions. The expression trend for both *TET2* and *DNMT3A* under Mac-CM *in vitro* was down-regulated compared to DM. Statistical analysis by one-way ANOVA with post-hoc Tukey * $P< 0.05$ (vs. Undifferentiated); # $p< 0.02$ (DM vs. Mac-CM).

a *In vitro* paradigm: Fig.2-Fig.7

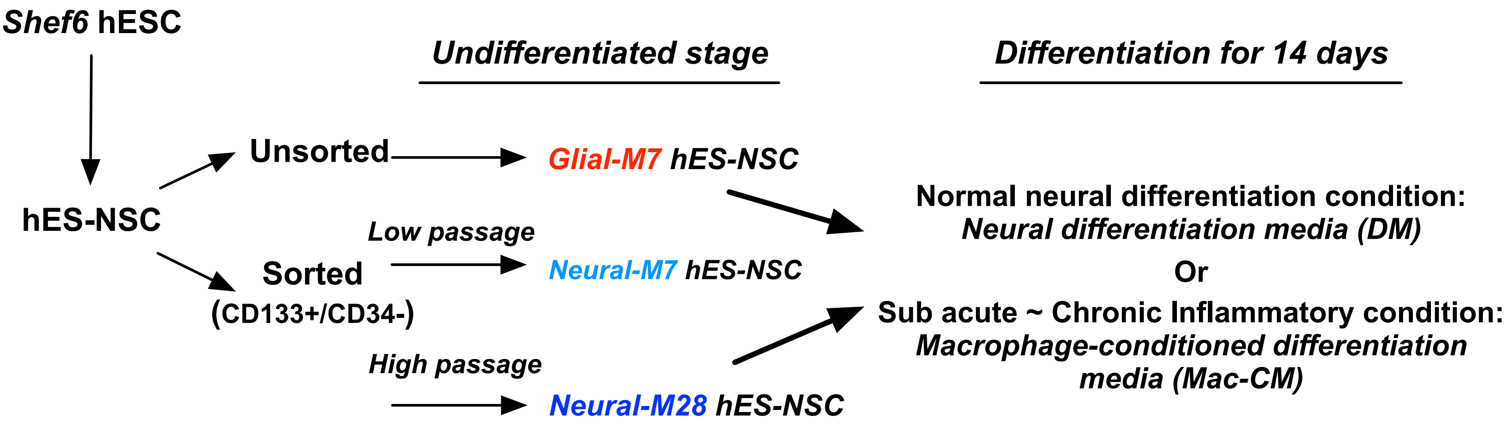

Detection	Method
mRNA	RNA-seq/qPCR
5-Methylcytosine (5mC)	RRBS
5-Hydroxymethylcytosine (5hmC)	RRHP

Method
qPCR
RRBS
RRHP

b *In vivo* paradigm: Fig.8

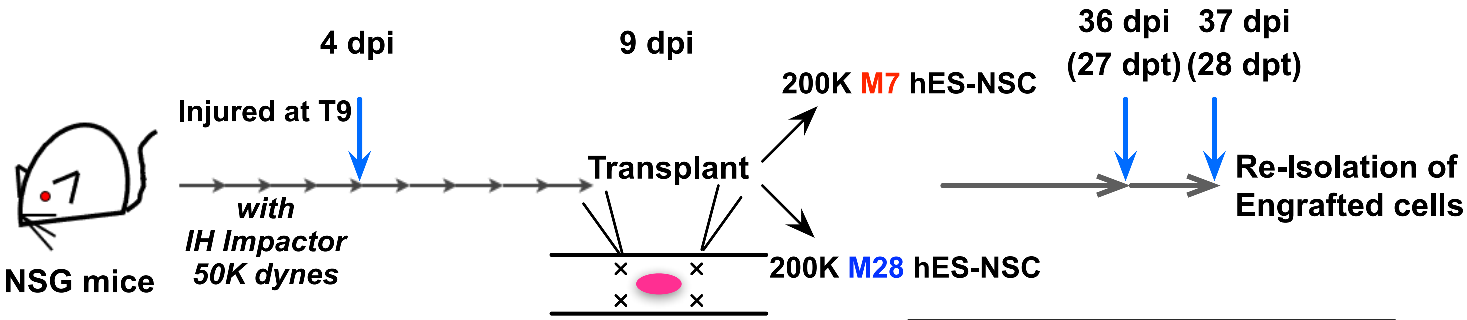

Detection	Method
mRNA	RNA-seq
5-Methylcytosine (5mC)	RRBS

Supplementary Figure S7

Epigenetic analysis for *in vitro* and *in vivo* paradigm overview. (**a**): *in vitro* paradigm (Fig. 2-7) and (**b**): *in vivo* paradigm (Fig. 8) are shown. Detection methods used in this study are shown in the box (qPCR and/or RNA-seq, RRBS, RRHP).

We analyzed undifferentiated hES-NSC, Glial-M7, Neural-M7, and Neural-M28, and after 14 days of differentiation. *In vitro* differentiation was performed under normal neural differentiation (DM) and Macrophage-enriched inflammatory (Mac-CM) conditions. In the *in vivo* study, each mouse's locomotor scale was checked using the BMS (Basso mouse scale) score to evaluate each animal and if the animal received the same injury level at 4 days post-injury (4 dpi) and transplantation (Tx) of either Glial-M7-GFP or Neural-M28-GFP hES-NSC was done at 9 days post spinal cord injury (9 dpi). We recovered each engrafted cell (either Glial-M7-GFP or Neural-M28-GFP) at 4 weeks post-transplantation (28 dpt) from the injured animal's spinal cord. One day before harvesting *in vivo* engrafted cells, we checked the locomotor scale of each mouse by BMS (Basso mouse scale for locomotion) score and we selected each mouse that got the same injury levels and recovery for this study.

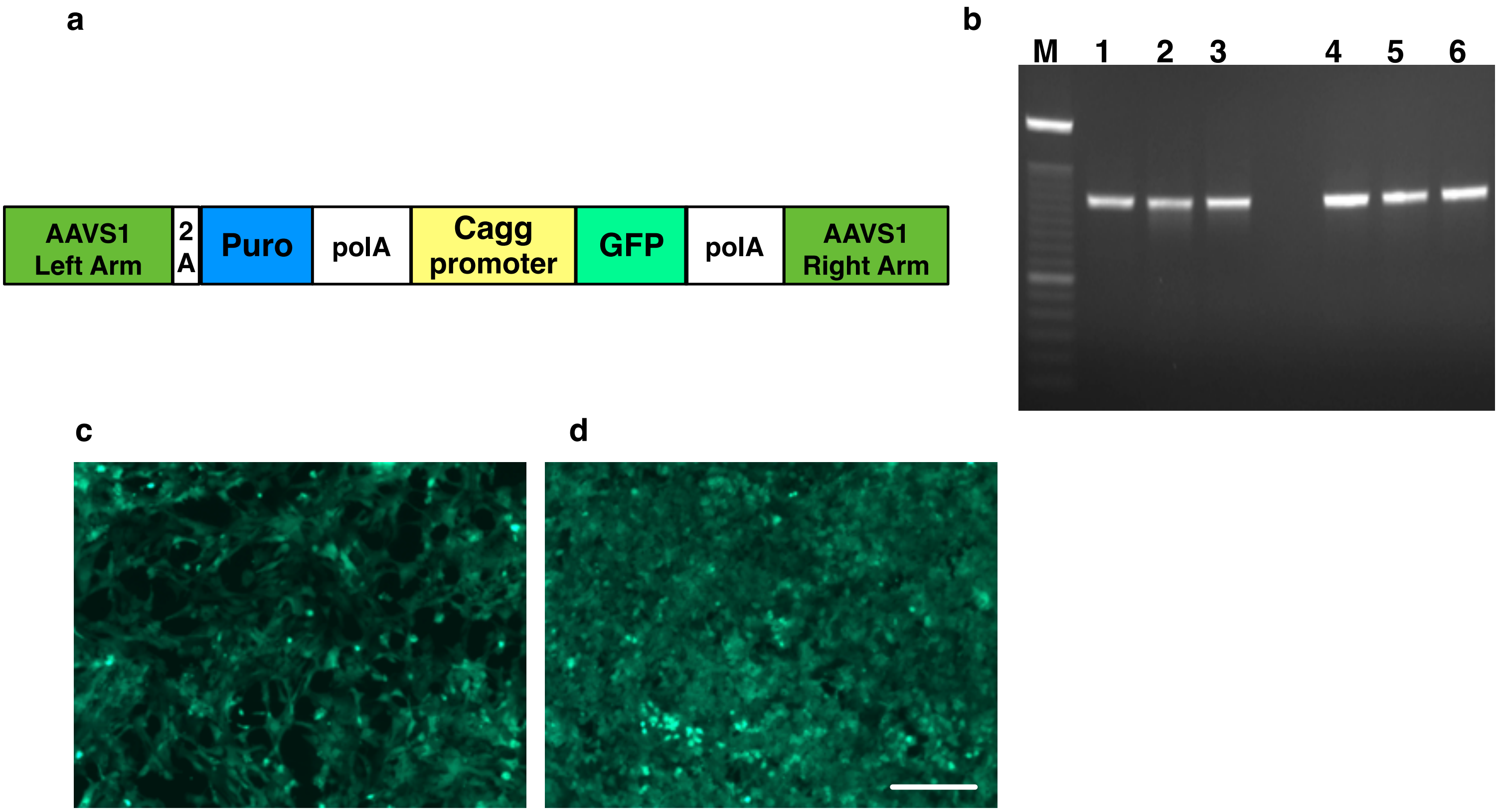

Supplementary Figure S8

Glial-M7 and Neural-M28 hES-NSC used for *in vivo* transplantation experiments.

Each cell line was engineered for GFP-reporter gene expression.

(**a**) This scheme shows the target gene of interest; Cagg promoter-GFP cassette that we cloned into pZDonor-AAVS1 puromycin vector (Sigma-Aldrich). (**b**) We confirmed the single junction amplicon band (approximately 1,100 bp) by PCR using AAVS1 forward, and Puro reverse primers (Sigma-Aldrich) after we performed targeted integration with the plasmid DNA shown in (**a)** and ZFN (Zinc finger nuclease) mRNA by transfection via Nucleofection (Lonza) to each hES-NSC either Glial-M7 or Neural-M28 along with both manufacturers' recommendations. We selected GFP expressing hES-NSC for parental hES-NSC of both Glial-M7 and Neural-M28 lines with puromycin selection. The target integration site is at the safe harbor loci; adeno-associated virus integration sites 1 (AAVS1) on human chromosome 19. Inverted microscope Image of Glial-M7-GFP (**c**) and Neural-M28-GFP (**d**) by Olympus IX71. The scale shown is 200 μm.

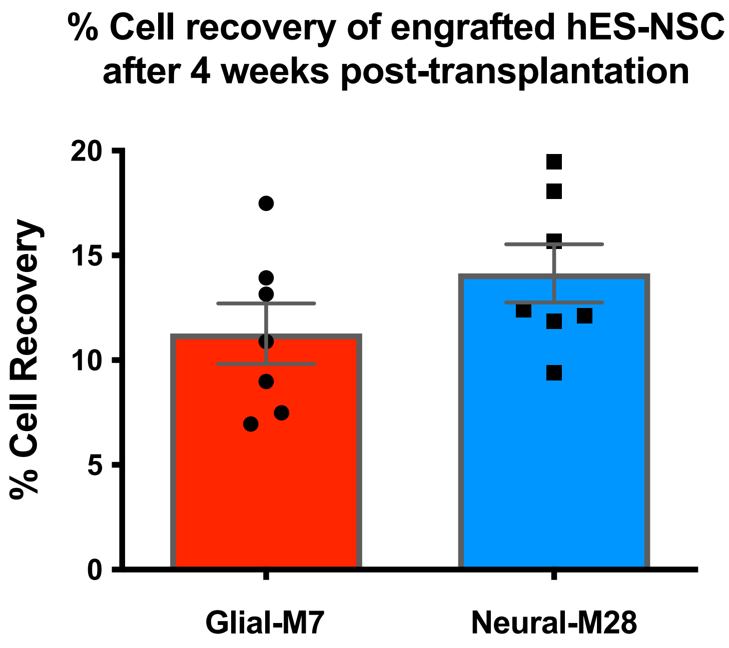

Cell line	Transplanted (Tx) cells/ animal	Putative Total Tx cells (4 animals/Replicate)	Biological Replicates	Average Number of Cell Recovery at 4 weeks/ Replicate	% Average Cell Recovery/Replicate
Glial-M7	200,000	800,000	7	102,546	12.8
Neural-M28	200,000	800,000	7	113,171	14.1

Supplementary Figure S9

Cell recovery of engrafted Glial-M7 or Neural-M28 hES-NSC after four weeks-post transplantation at injured SC. About 200,000 cells of Glial-M7-GFP or Neural-M28-GFP per mouse at 9 days post injury (dpi) were transplanted into the injured spinal cord. Engrafted cells were recovered from four pooled animal's injured spinal cords (as a biological replicate) at 4 weeks post-transplantation by FAC sorting (FACS). Each dot shows % cell recovery from each biological replicate by FACS. Each engrafted group; either Glial-M7 or Neural-M28 has seven biological replicates.

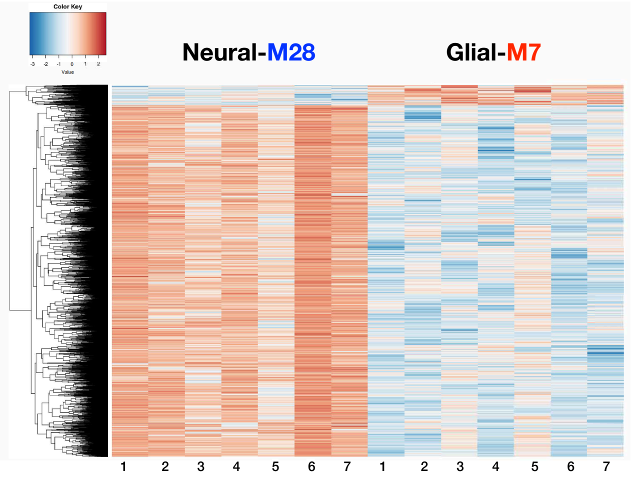

Supplementary Figure S10

Hierarchical clustering results of RNA-seq between *in vivo* re-isolated engrafted Neural-M28 and Glial-M7 cells from injured NSG mouse spinal cord are shown. The seven columns on the left show seven biological replicates for Neural-M28 and the next seven columns on the right show seven biological replicates for Glial-M7.

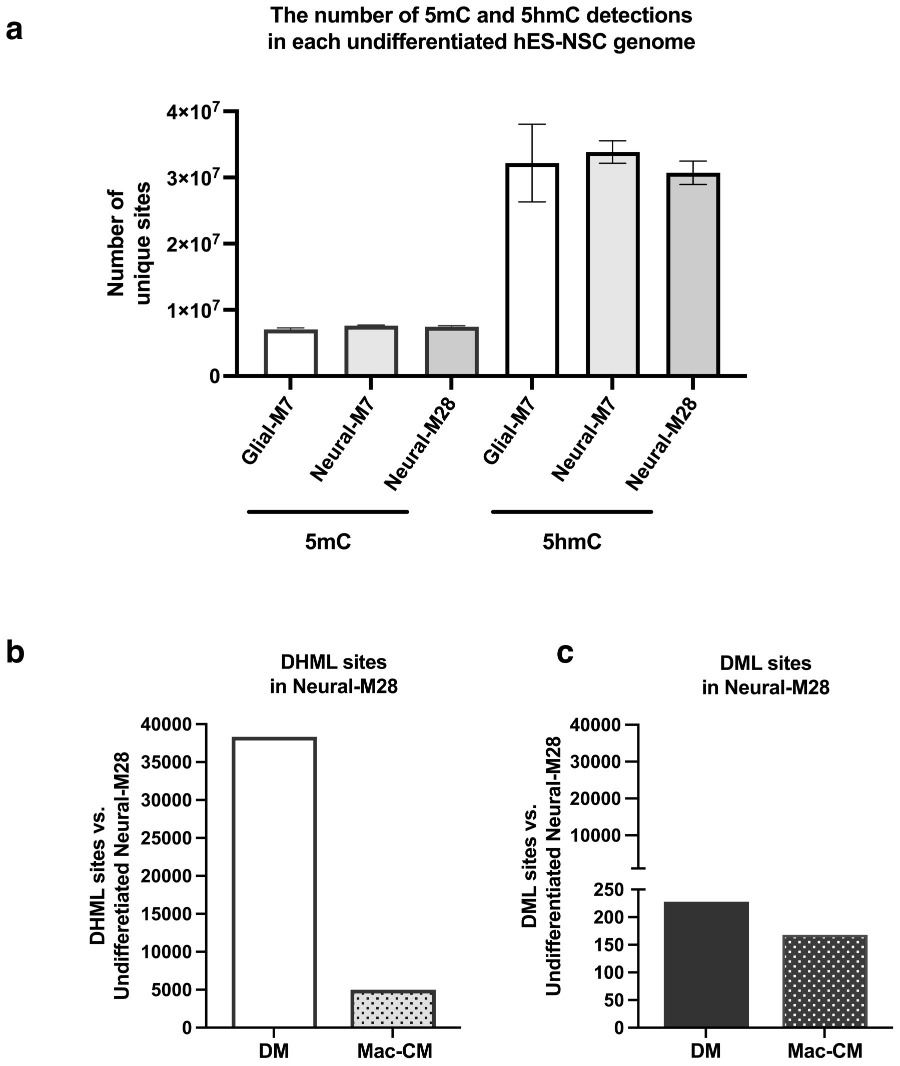

Supplementary Figure S11

Correlation between *TET2* gene expression and the increased number of 5hmC detections in the genome.

Graph (**a**) shows the number of unique 5mC and 5hmC sites detected by RRBS or RRHP in each undifferentiated hES-NSC genome (Glial-M7, Neural-M7, Neural-M28). The number of 5hmC sites may potentially be due to TET2 function during the transition from pluripotent stem cells. This result correlates to a degree with the results shown in Fig. 2g. Despite cell batch variability that exists, *TET2* gene expression levels and an increase in 5hmC detection numbers appears to correlate.

Graphs show the number of differential hydroxymethylation loci (DHML) (**b**) and differential methylation loci (DML) (**c**) detected during the 14-day differentiation under normal (DM) and macrophage-enriched inflammatory condition (Mac-CM) in Neural-M28 compared with undifferentiated Neural-M28.
Directional changes in *TET2* gene expression levels observed in Neural-M28 during differentiation under the two conditions, DM and Mac-CM (Fig. 7d and Supplementary Fig. S6) appears to correlate to an increase in 5hmC number detected.

Supplementary Tables S1. List of primer sequences for Taqman assay, regular qPCR, and shRNA target sequence for KD experiments.

Target gene	Taqman assay primer (Thermo Fisher)
18S	Hs99999901_s1
TBP	Hs99999910_m1
PROM1 (CD133)	Hs01009250_m1
NES	Hs00707120_S1
PDGFRA	Hs00998018_m1
GALC	Hs01012300_m1
CSPG4	Hs00361541_g1
CNP	Hs00263981_m1
MBP	Hs00921945_m1
S100B	Hs00902901_m1
GFAP	Hs00909236_m1
ALDH1L1	Hs00201836_m1
TUBB3	Hs00964962_g1
DCX	Hs00167057_m1
SYN1	Hs00199577_m1
MKI67	Hs01032443_m1

Gene	Forward(5'-)	Reverse(5'-)
18S rRNA	GTAACCCGTTGAACCCCATT	CCATCCAATCGGTAGTAGCG
DNMT1	AGAACGGTGCTCATGCTTACA	CTCTACGGGCTTCACTTCTTG
DNMT3A	AGTACGACGACGACGGCTA	CACACTCCACGCAAAAGCAC
DNMT3B	ACCTCGTGTGGGGAAAGATCA	CCATCGCCAAACCACTGGA
TET1	CATCAGTCAAGACTTTAAGCCCT	CGGGTGGTTTAGGTTCTGTTT
TET2	GGCTACAAAGCTCCAGAATGG	AAGAGTGCCACTTGGTGTCTC
TET2 tv1 (GeneCopoeia; HQP064384)	CGTCCATTCTCAGGGGTCAC	GTGCATACCAATGTGCTGCC
TET2 tv2 (GeneCopoeia; HQP013696)	ACCATAGGCAGTCTAATGTACGA	GGAGCACTAAGAGTGTCAGCA
TET3	GCCGGTCAATGGTGCTAGAG	CGGTTGAAGGTTTCATAGAGCC

shRNA (psi-LVRN1Mp Vector)		Target sequence (GeneCopoeia)
TET2	shRNA-1	gccttgaatgaagagagaact
	shRNA-3	ggtgaacatcattcaccttct
	shRNA-5	ccagggaaccacaaagcta
	shRNA-6	gaaattgaaacaagaccaa
	shRNA-8	caaaggtacttgatacata
DNMT3A	shRNA-1	gagacggcaaattctcagt
	shRNA-2	tgtgcggaaacaacaactg
	shRNA-3	taaccacgaccaggaattt
	shRNA-4	aagtgaggaccattactac
